# Distinguishing Optical and Acoustic Phonon Temperatures and Their Energy Coupling Factor under Photon Excitation in nm 2D Materials

**DOI:** 10.1002/advs.202000097

**Published:** 2020-05-26

**Authors:** Ridong Wang, Hamidreza Zobeiri, Yangsu Xie, Xinwei Wang, Xing Zhang, Yanan Yue

**Affiliations:** ^1^ State Key Laboratory of Precision Measuring Technology and Instruments Tianjin University Tianjin 300072 P. R. China; ^2^ Department of Mechanical Engineering Iowa State University Ames IA 50011 USA; ^3^ College of Chemistry and Environmental Engineering Shenzhen University Shenzhen Guangdong 518055 P. R. China; ^4^ Key Laboratory for Thermal Science and Power Engineering of Ministry of Education Department of Engineering Mechanics Tsinghua University Beijing 100084 P. R. China; ^5^ School of Power and Mechanical Engineering Wuhan University Wuhan 430072 P. R. China

**Keywords:** 2D materials, acoustic phonon temperature, energy coupling, energy transport state‐resolved Raman, optical phonon temperature

## Abstract

Under photon excitation, 2D materials experience cascading energy transfer from electrons to optical phonons (OPs) and acoustic phonons (APs). Despite few modeling works, it remains a long‐history open problem to distinguish the OP and AP temperatures, not to mention characterizing their energy coupling factor (*G*). Here, the temperatures of longitudinal/transverse optical (LO/TO) phonons, flexural optical (ZO) phonons, and APs are distinguished by constructing steady and nanosecond (ns) interphonon branch energy transport states and simultaneously probing them using nanosecond energy transport state‐resolved Raman spectroscopy. Δ*T*_OP −AP_ is measured to take more than 30% of the Raman‐probed temperature rise. A breakthrough is made on measuring the intrinsic in‐plane thermal conductivity of suspended nm MoS_2_ and MoSe_2_ by completely excluding the interphonon cascading energy transfer effect, rewriting the Raman‐based thermal conductivity measurement of 2D materials. *G*_OP↔AP_ for MoS_2_, MoSe_2_, and graphene paper (GP) are characterized. For MoS_2_ and MoSe_2_, *G*_OP↔AP_ is in the order of 10^15^ and 10^14^ W m^−3^ K^−1^ and *G*_ZO↔AP_ is much smaller than *G*_LO/TO↔AP_. Under ns laser excitation, *G*_OP↔AP_ is significantly increased, probably due to the reduced phonon scattering time by the significantly increased hot carrier population. For GP, *G*_LO/TO↔AP_ is 0.549 × 10^16^ W m^−3^ K^−1^, agreeing well with the value of 0.41 × 10^16^ W m^−3^ K^−1^ by first‐principles modeling.

## Graphical abstract

Upon photon excitation of 2D materials, the temperatures of optical and acoustic phonons are distinguished by constructing different interphonon energy transport states. A breakthrough is made on measuring the intrinsic in‐plane thermal conductivity of 2D materials by excluding interphonon branch thermal nonequilibrium. The phonon branch energy cooping factor is determined and agrees well with theoretical prediction.

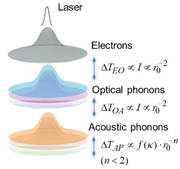

## 1 Introduction

As a powerful tool for characterizing 2D materials, Raman spectroscopy has been widely used to measure the thermal properties of 2D materials, e.g., graphene,^[^ ^1^, ^2^ ^]^ molybdenum disulfide (MoS_2_),^[^ ^3^, ^4^, ^5^ ^]^ molybdenum diselenide (MoSe_2_),^[^ ^3^ ^]^ etc. Many different Raman‐based methods, such as optothermal Raman technique,^[^ ^6^ ^]^ two‐laser Raman thermometry,^[^ ^7^ ^]^ variable‐spot‐size laser‐flash Raman method,^[^ ^8^ ^]^ time‐domain differential Raman (TD‐Raman) technique,^[^ ^9^ ^]^ frequency‐resolved Raman (FR‐Raman) spectroscopy,^[^ ^10^ ^]^ frequency‐domain energy transport state‐resolved Raman (FET‐Raman) technique,^[^ ^11^ ^]^ picosecond energy transport state‐resolved Raman (ps ET‐Raman) technique,^[^ ^12^, ^13^, ^14^ ^]^ and nanosecond energy transport state‐resolved Raman (ns ET‐Raman) technique, have been developed.^[^ ^15^ ^]^

Among these Raman‐based methods, the optothermal Raman technique is straightforward and is widely used to measure the thermal conductivity of 2D materials.^[^ ^1^, ^3^ ^]^ This technique uses the Raman spectrum to probe the temperature rise of the sample that is heated up by the Raman excitation laser. The two‐laser Raman thermometry method instead measures the temperature rise of the sample heated by a laser different from the Raman excitation laser.^[^ ^7^ ^]^ By varying the laser spot size, the laser‐flash Raman method could determine the thermal conductivity, thermal diffusivity, and interfacial thermal conductance of suspended or supported 2D materials.^[^ ^8^ ^]^ It uses a continuous wave (CW) laser and a pulsed laser to probe the transient thermal response of the sample. For transient thermal response detection, the TD‐Raman employs an amplitude‐modulated laser with varying heating cycle time and fixed cooling time, and provides a novel way to measuring thermal diffusivity with very high accuracy.^[^ ^9^ ^]^ In the FR‐Raman spectroscopy, a laser is modulated with a square‐wave for heating and simultaneous Raman‐based thermal probing. It features great accuracy and ease of implementation and has been used to measure the anisotropic thermal conductivity of suspended black phosphorus.^[^ ^10^ ^]^ The FET‐Raman is similar to the FR‐Raman, but only uses a single modulation frequency and measures the Raman shift change against the laser heating power.^[^ ^11^ ^]^ It makes significant advances over FR‐Raman in terms of measurement accuracy and feasibility. The ps ET‐Raman and ns ET‐Raman probably represent the most advanced Raman techniques for characterizing the thermal transport in 2D materials. They are able to push the time scale down to ps and ns like the pump–probe technique, but take very different ways in energy transport construction and probing. These techniques have been used to pioneer the characterization of interface thermal resistance, hot carrier diffusion coefficient, and in‐plane thermal conductivity of supported 2D MoS_2_, MoSe_2_, and WS_2_.^[^ ^12^, ^15^, ^16^ ^]^

Regardless of the different Raman‐based methods used for characterizing the thermal properties of 2D materials, the physical process happening inside these methods is similar. This process consists of energy transfer among energy carriers, which include photons, electrons, and phonons. For phonons, there are three optical branches, including longitudinal optical (LO), transverse optical (TO), and flexural optical (ZO) branches. In addition, there are three acoustic branches: longitudinal acoustic (LA), transverse acoustic (TA), and flexural acoustic (ZA) branches. Sullivan et al.^[^ ^17^ ^]^ used a first‐principles‐based multitemperature model (MTM) to calculate the local temperatures of electrons, LO phonons, LA phonons, and ZA phonons inside the Raman laser spot. The results showed that the temperatures of these energy carriers were at nonequilibrium. Lu et al.^[^ ^18^ ^]^ also found such kind of nonequilibrium, especially the ZA phonons showed the largest nonequilibrium from other phonon branches. As ZA phonons were the main heat carriers in the heat conduction process, neglect of nonequilibrium between ZA and LO/TO phonons (the ones probed by Raman spectroscopy) could result in significant underestimation of thermal conductivity by using Raman‐based methods. To date, this interphonon branch thermal nonequilibrium has never been considered in thermal conductivity measurement of 2D materials using Raman spectroscopy, not to mention the quantitative determination of the energy coupling factor among phonon branches and distinguishing the optical and acoustic phonon temperatures.

In order to improve the accuracy of thermal conductivity measurement by using Raman‐based methods, it is of great importance to explore the temperature nonequilibrium among energy carriers in materials. Waldecker et al.^[^ ^19^ ^]^ introduced a nonthermal lattice model to describe nonequilibrium phonon distributions in aluminum, and this method may be applied to a range of materials. Tian et al.^[^ ^20^ ^]^ explored the contributions to thermal conductivity in bulk silicon of different phonons, which included LA, TA, LO, and TO phonons, by using first‐principles calculations. Pop et al.^[^ ^21^ ^]^ used a Monte Carlo model, which could distinguish the optical/acoustic and the longitudinal/transverse phonon branches, for electron transport in silicon. A strong equivalent temperature nonequilibrium of different phonon branches was found. Mittal and Mazumder^[^ ^22^ ^]^ also used the Monte Carlo method to study the role of different phonon branches on thermal conductivity of silicon thin films. By using an exact numerical solution of the phonon Boltzmann equation, Lindsay et al.^[^ ^23^ ^]^ found out that the lattice thermal conductivity of graphene is dominated by the flexural phonon modes, which was previously thought to be negligible. Falcão et al.^[^ ^24^ ^]^ discovered the thermal nonequilibrium between optical and acoustic phonons for silicon nanocrystals by using Raman spectroscopy. Ferrante et al.^[^ ^2^ ^]^ studied the phonon nonequilibrium properties in the presence of hot charge carriers in graphene by detecting the Raman response of graphene under ultrafast laser excitation. All these studies are mainly focused on silicon and graphene. To date there is no experimental work on distinguishing the optical and acoustic phonon temperatures and quantifying the interbranch nonequilibrium effect on thermal conductivity measurement of 2D transition metal dichalcogenides (TMDs), such as MoS_2_ and MoSe_2_. And, such effect could be significant and strongly hinder the understanding of energy transport in 2D TMDs.

In this work, we design and employ the ns ET‐Raman technique to explore the temperature nonequilibrium among different phonon branches. In the experiments, a suspended 55 nm thick MoS_2_ and a suspended 71 nm thick MoSe_2_ are investigated. A breakthrough is made in distinguishing and measuring the temperatures of optical phonons (OPs) and acoustic phonons (APs) for these materials. The energy coupling factors, for the first time, are also determined by using Raman spectroscopy. Furthermore, the energy coupling factor between OP and AP of graphene paper (GP) is also measured by using a CW laser in Raman experiments.

## 2 Results and Discussion

### 2.1 Cascading Energy Transport in 2D Materials under Laser Irradiation

**Figure 1:**
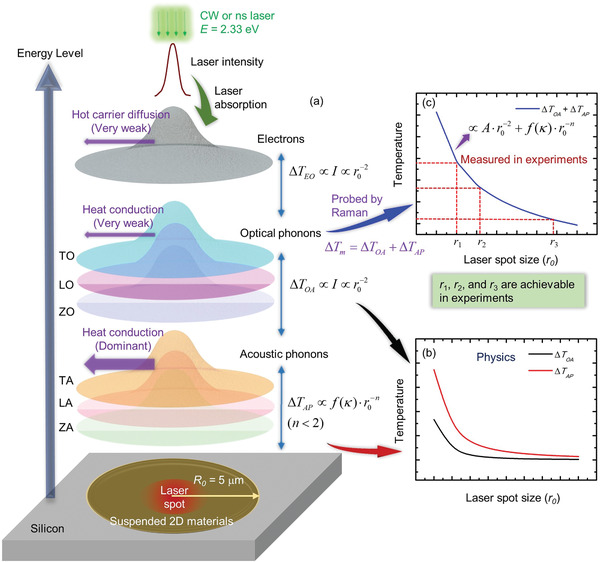
Illustration of the cascading energy transfer process among different energy carriers in 2D materials. a) A continuous wave (CW) or a nanosecond (ns) laser irradiates the suspended 2D material. With the laser absorption, the energy is transferred to electrons first. Then, the energy is mainly transferred from electrons to optical phonons. Next, the optical phonons will transfer most of the energy to acoustic phonons. Finally, the energy will be transferred to the whole area of the sample through heat conduction. b) The temperature difference between optical phonons and acoustic phonons decreases (≈$r_{0}^{-2}$) against increased laser spot size (*r*_0_). The temperature difference between acoustic phonons and environment also decreases with the increased laser spot size, but with a different rate. c) The thermal conductivity of the sample and the energy coupling coefficient between optical phonons and acoustic phonons can be determined by using three achievable laser spots in experiments (*r_i_*, *i* = 1, 2, 3). Note the temperature mentioned in the figures is a value at a point within the laser heating area for ease of physics discussion. In Raman experiment, the measured temperature is a Raman intensity‐weighted average, and is considered in our modeling and data processing.

First of all, we show the cascading energy transport in 2D materials upon photon excitation, and discuss the involved physics and the induced temperature rise probed by Raman spectroscopy. **Figure** **1**a shows the energy transfer process among different energy carriers, which provides the overall picture of the physical process studied in this work. First, subsequent to laser irradiation, electrons absorb the photon energy. Electrons will be excited to generate electron–hole pairs. Then electrons and holes (hot carriers) will diffuse and recombine, release the energy by scattering with OP. It typically takes nanoseconds for this diffusion process.^[^ ^13^ ^]^ Such hot carrier diffusion effect is more prominent for very small spot size laser irradiation. Our past work has firmly proved that for a suspended 2D material, the electron–hole diffusion has negligible effect on heat conduction. For instance, for a suspended WS_2_ of 10 µm diameter, the hot carriers only have an effect of ≈5% under 100× laser spot irradiation.^[^ ^16^ ^]^ Therefore it is physically reasonable to assume that the absorbed photon energy is transferred from hot carriers to OP rather than being conducted away by diffusion.

OP receives energy from hot carriers, and will have a prominent temperature rise. In the strict physical sense, they will transfer some energy to the substrate region via heat conduction in the suspended sample. Such energy transfer differs among the three optical phonon branches (TO, LO, and ZO). Due to the very small group velocity of OP and their very low specific heat, they have a relatively low thermal conductivity compared with that of acoustic phonons.^[^ ^20^, ^25^, ^26^, ^27^ ^]^ OP will transfer the majority of energy to AP through the energy coupling between these two kinds of phonons. This energy coupling process is also known as anharmonic coupling, which is a three‐phonon process in most of the cases. One OP can decay to two lower energy APs where the energy and momentum are conserved. This process can also happen inversely. The two APs have opposite momenta as the OP has zero momentum at the center of the Brillouin zone and in most of the cases both APs have similar energy.^[^ ^28^, ^29^, ^30^ ^]^ The anharmonic decay also determines the lifetime of these nonquilibrium phonons.^[^ ^31^ ^]^ Take MoS_2_ as an example, the phonon lifetime is about 38 ps.^[^ ^32^ ^]^ As will be detailed later, each OP branch will have different energy transfer to AP branches. Upon receiving energy from different OP branches, AP will transfer the energy to the edge of the suspended 2D material by heat conduction. However, this heat conduction is different for the three acoustic phonon branches (TA, LA, and ZA). In this work, we do not distinguish their difference, but instead use a lumped temperature and thermal conductivity to cover the effect of the three AP branches.

At a location *r* within the laser heating spot, the temperature difference between electrons and OP (Δ*T*_EO_) is the driving force behind the energy transfer from hot carriers to OP. Since the hot carrier diffusion has negligible effect on the energy distribution in the sample, it is safe to say that at any location under laser irradiation, the local Δ*T*_EO_ is proportional to the local absorbed laser energy as Δ*T*_EO_∝*I*. If the total laser energy is kept constant, we will have $I\propto r_{0}^{-2}$ where *r*_0_ is the laser spot size. The temperature difference between OP and AP (Δ*T*_OA_) is also the driving force behind the energy exchange between them. Due to the negligible heat conduction by OP, the energy transferred from hot carriers to OP will be transferred to AP before it is redistributed in space. So at any location of the sample, we will also have $\Delta T_{\mathrm{OA}}\propto I\propto r_{0}^{-2}$. Such physics base is firmly proved and discussed in Section 2.5. Note Δ*T*_OA_ here is not a specific one. In fact, it means the temperature difference between any OP branch and any AP branch, like Δ*T*_TO → ZA_, Δ*T*_LO → TA_, and so on.

The temperature rise of AP (Δ*T*_AP_) is related to both the laser spot size and the thermal conductivity of the 2D material. For the AP temperature, rather than distinguishing the temperature of each AP branch, we refer to a lumped AP average temperature. This is also entitled lattice temperature. The very strong heat conduction of AP weakens the dependence of Δ*T*_AP_ on the laser spot size. Therefore, we will have $\Delta T_{\mathrm{AP}}\propto f\left(\kappa \right)\cdot r_{0}^{-n}$ with *n* < 2. As shown in Figure 1b, the variations of Δ*T*_OA_ and Δ*T*_AP_ against laser spot size are different. When the laser spot is large enough, Δ*T*_OA_ approaches zero faster than Δ*T*_AP_. That is, the effect of energy transfer from OP to AP becomes negligible. In the Raman‐based temperature measurement, Raman peaks are corresponding to OP branches, which indicate that the probed temperature rise (Δ*T*_m_) is the temperature rise of OP. This temperature rise combines the effects of Δ*T*_OA_ and Δ*T*_AP_, and can be physically expressed as $\Delta T_{m}=\Delta T_{\mathrm{OA}}+\Delta T_{\mathrm{AP}}\propto Ar_{0}^{-2}+f\left(\kappa \right)\cdot r_{0}^{-n}$. When the laser spot is large enough, this temperature can be treated as the temperature of AP. Then, the determined thermal conductivity can be taken as the intrinsic one of the sample.

As shown in Figure 1c, Δ*T*_m_ can be measured under heating with different laser spot size (*r*_0_) (achievable using different objective lenses). The obtained Δ*T*_m_∼*r*_0_ data can be fitted using the function $Ar_{0}^{-2}+f\left(\kappa \right)\cdot r_{0}^{-n}$. As a result, we can determine: for the probed temperature rise, how much is contributed from Δ*T*_OA_ and how much is contributed from Δ*T*_AP_. Although *f* (*κ*) can be determined either numerically or analytically, the true thermal conductivity *κ* of the sample is needed in distinguishing Δ*T*_OA_ and Δ*T*_AP_. This is a critical part and will be achieved in this work to obtain a converged *κ* that truly reflects the thermal conduction capability of the sample. In this work, very much different from the widely used Raman method that needs the absolute laser absorption knowledge, Raman temperature coefficient, and absolute temperature rise, we will design an ns ET‐Raman technique to measure the true *κ* of the sample, distinguish Δ*T*_OA_ and Δ*T*_AP_, and determine the energy coupling factors among phonon branches. This ns ET‐Raman technique is completely free of any need of laser absorption data and absolute temperature rise, and provides the highest‐degree energy transport probing. Though the pulse width of the nanosecond (ns) laser is much larger than the phonon lifetime, we still can distinguish Δ*T*_OA_ and Δ*T*_AP_ based on their different responses to laser spot size variation. Note in our above physics description, we take the temperature at a location within the heating area for discussion. In real Raman experiment, the probed temperature is a Raman‐intensity weighted average. This is rigorously taken into consideration in our data processing.

### 2.2 ns ET‐Raman: Consideration of OP–AP Energy Transfer

**Figure 2:**
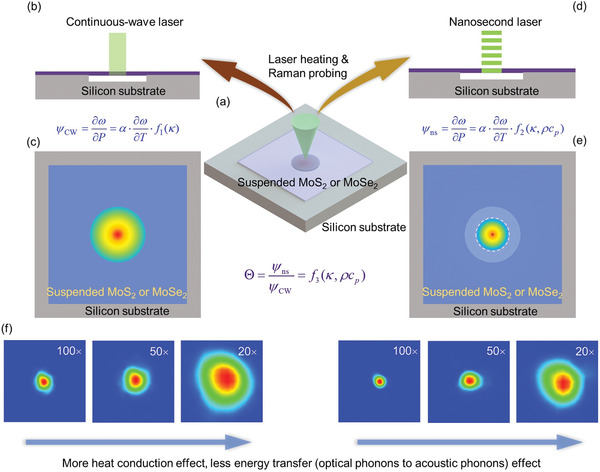
Physical concept of the ns ET‐Raman technique. a) MoS_2_ or MoSe_2_ nanosheets are transferred onto the silicon substrate with a hole beneath. b,d) A CW laser and an ns laser with the same wavelength (532 nm) are used to generate two energy transport states in the time domain. The laser is used for both heating the sample and probing the Raman signal. c,e) Heating effect of CW laser and ns laser. Based on the different contribution of in‐plane thermal conductivity under the two energy transport states, the in‐plane thermal conductivity of the sample could be obtained. f) With the increase of laser spot size, the effect of energy transfer from optical phonons to acoustic phonons decreases. The size of all the panes in (f) is 4.9 µm × 4.9 µm.

In the ns ET‐Raman technique, two different energy transport states in the time domain are constructed to probe the material's thermal response. The two probed energy transport states are steady state heating and transient state heating. As shown in **Figure** **2**a, the suspended sample is irradiated by the laser for both heating and Raman probing. The sample will absorb the laser energy and transport it along in‐plane and crossplane directions. As the lateral size is much larger than the sample thickness, the energy transport in the crossplane direction is negligible. Thus, the temperature distribution in this direction can be taken uniform. Figure 2b,c shows that a CW laser is used to generate steady state heating, and to excite Raman signal. The excited Raman signal can be collected to probe the temperature change of the sample. By using different laser powers (*P*), a parameter termed Raman shift power coefficient (*ψ*) is obtained as *ψ*_CW_ = ∂*ω*/∂*P* = *α* · (∂*ω*/∂*T*) · *f*_1_(*κ*_∥_), where *α* is laser absorption coefficient, ∂*ω*/∂*T* the temperature coefficient of Raman shift, and *κ*_∥_ the in‐plane thermal conductivity of the sample. As shown in Figure 2d,e, a ns laser is used to generate transient state heating and probe the Raman signal emitted during the ns pulse. The probed Raman shift change reflects the temporal and spatial averaged thermal response of the sample. Similarly, we have *ψ*_ns_ = ∂*ω*/∂*P* = *α* · (∂*ω*/∂*T*) · *f*_2_(*κ*_∥_,*ρc*_p_), where *ρc*_p_ is volumetric heat capacity of the sample. Comparing the two energy transport states, the effect of heat conduction is highly related to in‐plane thermal conductivity of the sample and is different for *ψ*_CW_ and *ψ*_ns_. Under transient state, the thermal diffusion length from the heating region is much smaller than that under steady state.

Based on *ψ*_CW_ and *ψ*_ns_, a dimensionless *ψ* is defined as Θ = *ψ*_ns_/*ψ*_CW_ = *f*_3_(*κ*_∥_,*ρc*_p_). The effects of *α* and ∂*ω*/∂*T* are completely canceled out in *Θ*. In the experiments, very low laser powers are chosen to ensure a moderate temperature rise of the sample. As a result, the temperature effect on *ρc*_p_ and *κ*_∥_ variation with *T* can be neglected. Consequently, *Θ* is only related to in‐plane thermal conductivity of the sample. A 3D heat conduction model is used to simulate the temperature distributions under the two energy transport states. Based on this, a theoretical curve between the temperature rise ratio of the two states and in‐plane thermal conductivity of the sample can be obtained. *κ*_∥_ of the sample can be finally determined by interpolating the measured *Θ* in the theoretical curve. Our previous study has firmly proved this technique can measure *κ*_∥_ of 2D materials with high accuracy.^[^ ^15^ ^]^

Note in the experimentally obtained Θ = *ψ*_ns_/*ψ*_CW_, the measured *ψ*_CW_ and *ψ*_ns_ in fact both are not only determined by heat conduction, but also include the effect of OP to AP energy transfer. That is, we have $\Theta ={\left[Ar_{0}^{-2}+f\left(\kappa_{∥}\right)\cdot r_{0}^{-n}\right]}_{\mathrm{ns}}/{\left[Ar_{0}^{-2}+f\left(\kappa_{∥}\right)\cdot r_{0}^{-n}\right]}_{\mathrm{CW}}$. Therefore, the determined *κ*_∥_ is not the intrinsic thermal conductivity of the sample, rather an effective value: *κ*_eff_. To determine the intrinsic *κ*_∥_, the experiments are conducted using different *r*_0_ to explore the variation of *κ*_eff_ with *r*_0_. It is expected that with the increase of laser spot size, the effect of energy transfer from optical phonons to acoustic phonons is diminishing. By studying this diminishing trend, we can finally determine the intrinsic *κ*_∥_. In this work, as shown in Figure 2f, three objective lenses (100×, 50×, and 20×) are used to vary the laser spot size for both CW and ns lasers.

**Figure 3:**
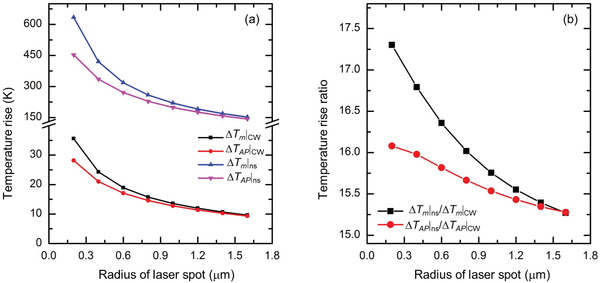
Hypothetical illustration of temperature rise variation against laser spot size. The temperature rise (Δ*Τ*_m_) obtained from the ns ET‐Raman is attributed to the energy transfer from optical phonons to acoustic phonons (Δ*Τ*_OA_) and the heat conduction of acoustic phonons (Δ*Τ*_AP_). a) Temperature rise variation with the laser spot size under CW laser and ns laser heating. Under ns laser, there is also an effect from the specific heat since the heat transfer does not reach the steady state yet. b) Temperature rise ratio variation against the laser spot size by ns ET‐Raman and simulation.

To clearly show the physics of the converged (intrinsic) *κ*_∥_ determination, **Figure** **3**a shows the variation of hypothetical temperature rise (Raman intensity weighted) and true acoustic phonon temperature rise under different laser spot sizes for MoS_2_ under CW and ns cases with constant laser power heating. As shown in this figure, the Raman measured temperature rise will decrease with the increased laser spot size for both CW and ns cases. As the laser intensity also changes with the laser spot size, the contributions of AP (Δ*T*_AP_) and energy transfer from OP to AP (Δ*T*_OA_) will change. For both CW and ns cases, the contribution of Δ*T*_OA_ can be quite negligible when the radius of the laser spot is larger than 1.2 µm. The temperature rise (Δ*T*_m_) obtained from ns ET‐Raman experiments is attributed to both Δ*T*_AP_ and Δ*T*_OA_, while the temperature rise obtained from the 3D heat conduction model is only related to Δ*T*_AP_. In Figure 3b, *Θ*, which is based on Δ*T*_m_, is plotted out and compared with the ratio of Δ*T*_AP_|_ns_/Δ*T*_AP_|_CW_. When the laser spot size goes bigger, *Θ* converges to the value of Δ*T*_AP_|_ns_/Δ*T*_AP_|_CW_, and reflects the true effect of phonon heat conduction.

### 2.3 Thermal Conductivity Convergence of MoS_2_

First of all, before we try to distinguish and probe the temperature rise of OP and AP in MoS_2_, its intrinsic thermal conductivity is measured, which is needed for later on data processing. Such measurement provides unprecedented data over documented work that do not consider the effect of OP−AP energy transfer. As shown in Figure S1a in the Supporting Information, the Raman spectrum of MoS_2_ has two vibrational modes ($E_{2g}^{1}$ and A_1g_). The $E_{2g}^{1}$ mode, which is related to LO and TO phonons, is associated with the opposite vibration of two sulfur atoms with respect to the molybdenum atom in the in‐plane direction. The A_1g_ mode, which is related to ZO phonon, is associated with the opposite vibration of sulfur atoms in the crossplane direction.^[^ ^33^ ^]^ Both modes can be used to characterize the thermal properties of MoS_2_.^[^ ^3^, ^4^, ^5^, ^12^, ^34^ ^]^

The ns ET‐Raman experiments are conducted by using three objective lenses (20×, 50×, and 100×). Room temperature Raman spectra are collected automatically under different laser powers to obtain *ψ*. The laser powers used for the MoS_2_ sample are listed in **Table** **1**. More experimental details can be found in our past work.^[^ ^15^, ^16^ ^]^ The radii of the laser spots are also measured and listed in Table 1. The spot size difference between the two lasers under the same objective lens mainly comes from the difference of their collimation level. The phonon mean free path of MoS_2_ is around 15 nm,^[^ ^35^ ^]^ while the radius of the laser spot in the experiments is about 300 nm or larger. Thus, the thermal transport can be safely taken as diffusive and local equilibrium.^[^ ^32^ ^]^

**Table 1:** Summary of CW and ns laser powers for 55 nm thick MoS_2_ and the corresponding laser spot radii

Objective lens	CW laser power range [mW]	ns laser power range [mW]	CW laser spot radius [µm]	ns laser spot radius [µm]
20×	1.39–6.72	0.16–0.76	1.355	1.060
50×	0.61–2.97	0.10–0.47	0.625	0.493
100×	0.50–2.40	0.07–0.36	0.405	0.311

**Figure 4:**
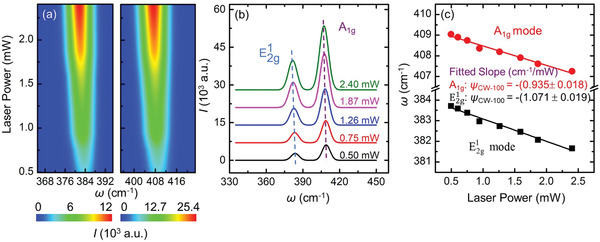
a) 2D contour map of MoS_2_ Raman peaks. This figure demonstrates the variation of Raman shift against the laser power of CW laser with 100× objective lens. b) Five representative Raman spectra of MoS_2_ with increased laser power of CW laser with 100× objective lens. Both modes are redshifted with increased laser power. For CW laser with 100× objective lens, the Raman shifts of the two modes as a function of laser power are shown in (c).

**Figure** **4**a shows the 2D contour map of the Raman peaks of MoS_2_ under CW laser with 100× objective lens. The two Raman peaks are observed to redshift with the increased laser power. Five representative room temperature Raman spectra under CW laser are shown in Figure 4b. The results also confirm that the two Raman peaks are redshifted with the increased laser power. That is, the local temperature of the sample is increasing with the increased laser power. As shown in Figure 4c, there is a good linear relationship between the positions of two peaks and the laser power. It is noted although the experimental spectral resolution is around 1.2 cm^−1^ for the measured spectrum, the determined Raman shift has an uncertainty of less than 0.03 cm^−1^ after fitting. The results of MoS_2_ with other two objective lenses and the results under ns laser are shown in Figures S2 and S3 in the Supporting Information, respectively. Similar observations are made as that for the CW Raman results as shown in Figure 4. All the obtained *ψ* values are listed in **Table** **2**. The *ψ* values decrease with the increased laser spot size for both CW and ns laser heating. For a larger spot size, the temperature rise of the sample is smaller due to the more spatially distributed laser energy.

**Table 2:** Summary of *ψ* values under the two lasers for different objective lenses

Objective lens	*ψ*_CW_ [cm^−1^ mW^−1^]		*ψ*_ns_ [cm^−1^ mW^−1^]		*Θ*	
	$E_{2g}^{1}$	A_1g_	$E_{2g}^{1}$	A_1g_	$E_{2g}^{1}$	A_1g_
20×	−(0.435 ± 0.008)	−(0.379 ± 0.008)	−(3.415 ± 0.075)	−(2.969 ± 0.052)	7.85 ± 0.20	7.83 ± 0.20
50×	−(0.869 ± 0.014)	−(0.772 ± 0.007)	−(6.123 ± 0.132)	−(5.563 ± 0.120)	7.05 ± 0.18	7.20 ± 0.19
100×	−(1.071 ± 0.019)	−(0.935 ± 0.018)	−(7.968 ± 0.141)	−(7.500 ± 0.091)	7.43 ± 0.19	8.02 ± 0.21

The normalized *ψ* (*Θ*), which is the ratio of the two *ψ* values of CW and ns lasers with the same objective lens, is calculated for these results. These values are also summarized in Table 2. Then, 3D numerical modeling based on the finite volume method is conducted to calculate the temperature rise under the two energy transport states to determine the in‐plane thermal conductivity of MoS_2_ with different objective lenses. Note the measured *ψ* is not simply proportional to the local temperature rise, rather it is proportional to the Raman intensity‐weighted temperature in space (for CW case) and in time‐space (for ns case). All these are carefully considered in the data processing and numerical modeling. The laser power used in the modeling is very low (20 µW) to ensure a small temperature rise. The measured laser spot size is used in the modeling to guarantee the simulation accuracy.

**Figure 5:**
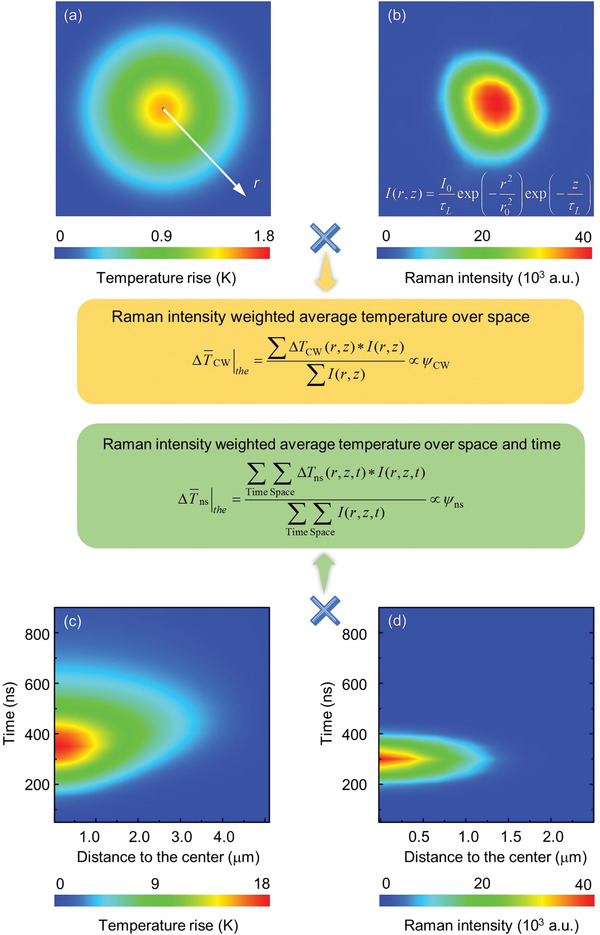
Physics of the measured temperature rise in ns ET‐Raman. a) Temperature distribution under CW laser heating. b) Raman intensity distribution of CW laser in space. c) Temperature map in the time‐space domain under ns laser heating. d) Raman intensity distribution of ns laser in time and space domains. The measured temperature rise is Raman intensity weighted over space domain in CW laser heating, and over time and space domains in ns laser heating. These two are proportional to the *ψ* values obtained in the ns ET‐Raman experiments. All these are considered in our 3D numerical modeling.

**Figure** **5**a,b shows the physics to calculate the Raman intensity‐weighted spatial average temperature rise under CW laser heating. A Raman intensity weighted average temperature rise over space ($\Delta {\bar{T}}_{\mathrm{CW}}{|}_{\mathrm{the}}$) is obtained, which is proportional to the corresponding *ψ*_CW_. Figure 5c,d shows the physics of calculating the Raman intensity‐weighted spatial and temporal average temperature rise. A Raman intensity weighted temperature rise over space and time ($\Delta {\bar{T}}_{\mathrm{ns}}{|}_{\mathrm{the}}$), which is also proportional to the corresponding *ψ*_ns_, is obtained. Then the ratios of these two values: ${\Theta |}_{\mathrm{the}}=\Delta {\bar{T}}_{\mathrm{ns}}{|}_{\mathrm{the}}/\Delta {\bar{T}}_{\mathrm{CW}}{|}_{\mathrm{the}}$ for different trial *κ*_eff_ values are used to determine the theoretical curve of *Θ* against *κ*_eff_ under the three objective lenses.

**Figure 6:**
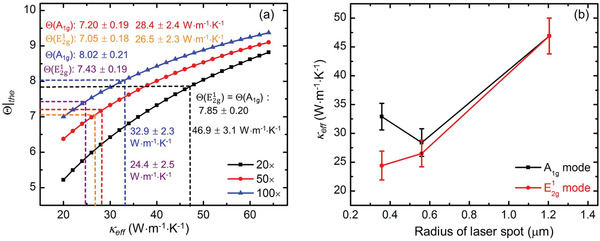
3D numerical modeling and data processing results for the 55 nm thick MoS_2_. The in‐plane *κ*_eff_ is obtained by interpolating the experimental results in the curves. a) In‐plane *κ*_eff_ obtained under the 20× , 50× , and 100× objective lenses for the two Raman modes. b) In‐plane *κ*_eff_ variation against laser spot size of the two Raman modes.

**Figure** **6**a shows that the *κ*_eff_ based on the two Raman modes that are obtained by interpolating the experimental results in the theoretical curves. All the obtained *κ*_eff_ values are summarized in Figure 6b. As shown in this figure, *κ*_eff_ obtained based on the $E_{2g}^{1}$ mode is increasing with the increased laser spot size. And, *κ*_eff_ obtained based on the A_1g_ mode decreases when the objective lens changes from 100× to 50×, and then increases when the objective lens changes from 50× to 20× . As the temperature nonequilibrium between OP and AP is highly related to the laser spot size, the nonmonotonic variance behavior of these two modes are mainly due to the laser spot size difference between the CW and ns lasers under the same objective lens. In addition, the experimental uncertainty also contributes to this nonmonotonic behavior. As shown in Figure 3a, the temperature rises of AP in ns ET‐Raman experiments decrease with the increased laser spot size for both CW laser and ns laser. The differences also decrease with the increased laser spot size. Figure 3b shows that bothΔ*T*_AP_|_ns_/Δ*T*_AP_|_CW_and Δ*T*_m_|_ns_/Δ*T*_m_|_CW_decrease with the increased laser spot size, which also indicates that the difference between OP and AP temperature rise decreases with the increased laser spot size. The difference between *κ*_eff_ when using the $E_{2g}^{1}$ and A_1g_ modes are caused by the different Δ*T*_OA_ since each OP branch has different level of energy coupling with the acoustic phonons. For the three OP branches, the temperature rises of LO and TO phonons are usually much larger than that of ZO phonons.^[^ ^18^ ^]^ This makes the measured *Θ* using the $E_{2g}^{1}$ peak is smaller than that using the A_1g_ peak. As a result, *κ*_eff_ values obtained based on the A_1g_ mode are larger than these values obtained based on $E_{2g}^{1}$ mode. With the increased laser spot size, the temperature rises of these three phonon branches are getting closer. The difference between the *κ*_eff_ values obtained based on the two Raman modes will also diminish. When a 20× objective lens is used, these two values converge to the same value, which is 46.9 ± 3.1 W m^−1^ K^−1^. This is the intrinsic *κ*_∥_ of 55 nm thick MoS_2_. In this work, *κ*_∥_ will be used not only in distinguishing temperatures of AP and OP, but also in evaluating the energy coupling factor between them in the next section.

Many previous studies have measured the in‐plane thermal conductivities of MoS_2_ with different thickness. For bulk MoS_2_, *κ*_∥_ is about 98.5 W m^−1^ K^−1^.^[^ ^36^ ^]^ For 4 and 7 layers thick suspended MoS_2_, *κ*_∥_ values are about 47 and 50 W m^−1^ K^−1^, respectively.^[^ ^37^ ^]^ As there is an increasing trend for *κ*_∥_ with the increased thickness, *κ*_∥_ of 55 nm thick MoS_2_ should be larger than 50 and less than 98.5 W m^−1^ K^−1^. However, those reported work never ruled out the effect of OP–AP energy transfer. The quality of the prepared samples and the measurement methods can also affect the results.^[^ ^4^ ^]^ In addition, there is a large discrepancy among the obtained temperature coefficients of the samples in the reported works using optothermal Raman spectroscopy. Last, the large difference among the laser absorption coefficients used in the reported work also affected the measurement significantly.^[^ ^3^ ^]^ In summary, it is critically important to point out that when Δ*T*_OA_ is not negligible, the *κ*_∥_ measurement data (apparent, or effective *κ*_∥_) will vary, depending on which Raman mode to use, and what is the laser spot size used in experiments. Only the converged one reflects the intrinsic thermal conductivity of the sample. Unfortunately, this has never been addressed in the past.

### 2.4 Distinguish Temperatures of AP and OP and Determine Their Energy Coupling Factor

In ns ET‐Raman experiment, the measured *ψ* values are proportional to the Raman intensity weighted temperature rise of the sample. The temperature rise is related to both the AP and OP in the material. That is, the temperature rise at any specific spatial point consists of both Δ*T*_OA_ and Δ*T*_AP_, and can be written as

(1) $$\begin{array}{c} \Delta T_{m}=\Delta T_{\mathrm{AP}}+\Delta T_{\mathrm{OA}}=\Delta T_{\mathrm{AP}}+\delta I/G_{\mathrm{pp}} \end{array}$$

where *G*_pp_ is the energy coupling factor between OP and AP, *I* is the absorbed laser intensity of the laser at location *r*, and *δ* (0 < *δ* < 1) is portion of laser energy transferred from the measured Raman mode optical phonons to acoustic phonons. Here, we assume the energy transferred from hot carriers to the three optical branches are uniform for order analysis. As the $E_{2g}^{1}$ mode is related to both LO and TO branches, *δ* is taken as 2/3. The A_1g_ mode is only related to ZO branch, and the corresponding *δ* is taken as 1/3. As shown in Equation (1), Δ*T*_OA_ is proportional to the laser intensity. This will be justified in next section using graphene as an example as its energy coupling among phonon branches has been studied well. In this section, we take the CW laser heating case for data analysis. For CW laser, the laser intensity is expressed by

(2) $$\begin{array}{c} I_{\mathrm{CW}}=\left(I_{0}/\tau_{L}\right)\exp\left(-r^{2}/r_{0}^{2}\right)\exp\left(-z/\tau_{L}\right) \end{array}$$

where $I_{0}=P/\pi r_{0}^{2}$ is the absorbed laser power per unit area at the center of laser spot, *r*_0_ (µm) is the radius of laser spot, and *τ*_L_ is the laser absorption depth, which is equal to 36.5 nm for MoS_2_.^[^ ^38^ ^]^ Based on Equations (1) and (2), the Raman intensity weighted temperature rise measured under CW laser case can be written as

(3) $$\begin{array}{ccc} {\left.\Delta {\bar{T}}_{m}\right|}_{\mathrm{CW}} & = & \frac{\int \int {\left.\Delta T_{m}\right|}_{\mathrm{CW}}I_{\mathrm{CW}}e^{-z/\tau_{L}}2\pi rdrdz}{\int \int I_{\mathrm{CW}}e^{-z/\tau_{L}}2\pi rdrdz}\\ & = & {\left.\Delta {\bar{T}}_{\mathrm{AP}}\right|}_{\mathrm{CW}}+\frac{1}{4}\cdot \frac{I_{0}}{\tau_{L}}\cdot \frac{\delta }{{\left.G_{\mathrm{pp}}\right|}_{\mathrm{CW}}} \end{array}$$

**Figure 7:**
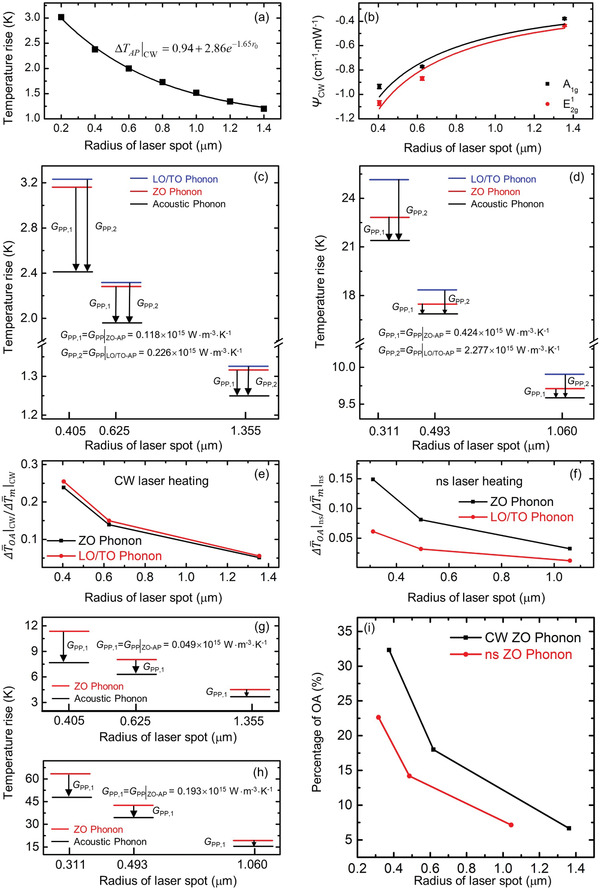
Determination of coupling factors of MX_2_ (MoS_2_ and MoSe_2_) under CW and ns laser heating. a) Theoretical temperature rise of acoustic phonons in MoS_2_ under CW laser heating. b) *ψ*_CW_ against laser spot size for MoS_2_. Distinguished temperatures of LO/TO phonon, ZO phonon, and AP under c) CW laser heating and d) ns laser heating with 20 µW absorbed laser power for MoS_2_. Contribution of Δ*T*_OA_ to the overall Δ*T*_m_ for LO/TO phonon and ZO phonon under e) CW laser heating and f) ns laser heating for MoS_2_. Distinguished temperatures of ZO phonon and AP g) under CW laser heating and h) ns laser heating with 20 µW absorbed laser power for MoSe_2_. i) Contribution of Δ*T*_OA_ to the overall Δ*T*_m_ for ZO phonon under CW laser heating and ns laser heating for MoSe_2_.

The term $e^{-z/\tau_{L}}$ is for the dissipation of the Raman signal when it transfers back to the sample surface.^[^ ^16^ ^]^ As *ψ*_CW_ obtained in Raman experiment is proportional to this temperature rise, the *ψ*_CW_∼*r*_0_ relation can be used to obtain *G*_pp_|_CW_. 3D numerical modeling based on the finite volume method is conducted to calculate the temperature rise under CW laser with different laser spot sizes. In this modeling, only acoustic phonons’ lumped thermal conductivity is considered, in order to obtain the acoustic phonon temperature rise under various laser heating condition. **Figure** **7**a shows the variation of $\Delta {\bar{T}}_{\mathrm{AP}}{|}_{\mathrm{CW}}$ against laser spot size in our modeling for the 55 nm thick MoS_2_. 20 µW absorbed laser irradiation is used in modeling, and the laser beam is assumed to have no reflection. Note such reflection treatment does not affect the data fitting and processing since the coefficient *A* used in Equation (4) has the reflection effect inside. In this modeling, the real thermal conductivity of the sample measured above is used. An exponential fitting method is used to fit the data to develop the relation under CW laser. The relation is also shown in Figure 7a and is expressed as $\Delta {\bar{T}}_{\mathrm{AP}}{|}_{\mathrm{CW}}=0.94+2.86e^{-1.65r_{0}}$. This relation reflects the Raman‐probed temperature rise, and shows how the overall acoustic phonon temperature rise is affected by the laser spot size. The relation between *ψ*_CW_ and $\Delta {\bar{T}}_{m}{|}_{\mathrm{CW}}$can be written as

(4) $$\begin{array}{c} \psi_{\mathrm{CW}}=A\cdot \left[\left(0.94+2.86e^{-1.65r_{0}}\right)+\frac{1}{4}\cdot \frac{P}{\pi r_{0}^{2}\tau_{L}}\cdot \frac{\delta }{{\left.G_{\mathrm{pp}}\right|}_{\mathrm{CW}}}\right]/P \end{array}$$

where *A* is determined by the Raman shift temperature coefficient and laser absorption. Here, *P* takes 0.02 mW, and the equation calculates the Raman shift change induced by 1 mW absorbed laser power. Then, the discovered relation between the acoustic phonon temperature rise and laser spot size is used to fit the variation of *ψ*_CW_ against laser spot size based on Equation (4). The *ψ*_CW_ values obtained under the three objective lenses and the fitting curves are shown in Figure 7b for the two Raman modes. The *A* values for $E_{2g}^{1}$ mode and A_1g_ mode are determined as −6.47 × 10^−3^ and −6.93 × 10^−3^ cm^−1^ K^−1^, respectively. At 532 nm, we estimate that the 55 nm thick MoS_2_ has a laser absorption of 40.5% (*n* = 5.238, *k* = 1.160). Therefore, based on *A*, the Raman temperature coefficient of MoS_2_ is estimated to be −0.016 and −0.0171 cm^−1^ K^−1^ for $E_{2g}^{1}$ and A_1g_ modes. These agree well with our previous directly determined Raman temperature coefficient of MoS_2_ at −0.0174 cm^−1^ K^−1^ ($E_{2g}^{1}$) and −0.0194 cm^−1^ K^−1^ (A_1g_) for 46 nm thick MoS_2_.^[^ ^39^ ^]^ This firmly justifies the validity of the data fitting here. Based on these fitting results, the energy coupling factors between OP and AP for the two modes under CW laser are obtained. *G*_pp_|_CW_ values for $E_{2g}^{1}$ mode and A_1g_ mode are determined as 0.226 × 10^15^ and 0.118 × 10^15^ W m^−3^ K^−1^, respectively.

Based on the fitting results from Figure 7b, the contribution of Δ*T*_OA_ and Δ*T*_AP_ to the measured Δ*T*_m_ can be distinguished from the Raman results. Figure 7c shows the determined temperatures of LO/TO phonon, ZO phonon, and acoustic phonon based on the data fitting for $E_{2g}^{1}$ and A_1g_ modes. The data is calculated based on 20 µW absorbed laser in the sample. It clearly shows the cascading energy transfer from OP to AP during laser heating. As shown in Figure 7c, the LO/TO phonon temperature is a little bit higher than that of the ZO phonon while both are much higher than that of AP. However, this temperature difference decreases with the increased laser spot size. That is, the contribution of Δ*T*_OA_ in the Raman‐measured temperature rise is decreasing with the increased laser spot size, which is shown in Figure 7e. Figure 7c also shows that the contributions of Δ*T*_OA_ for the two branches of optical phonons under CW laser are very close, which corresponds to their close *G*_pp_|_CW_ values. It is very important to note when the laser spot size is small (under 100× objective), the temperature difference between OP and AP takes a significant portion of the measured temperature rise (>25%). That is, if a smaller laser spot is used in Raman thermal conductivity measurement, neglecting the temperature difference between OP and AP will lead to a significantly underestimated thermal conductivity. The small temperature difference between LO/TO phonon and ZO phonon also indicates that the energy exchange between them during laser heating will be negligible compared with that between OP and AP, which has a much higher temperature difference as shown in Figure 7c.

Similarly, the energy coupling factors between OP and AP for the 55 nm thick MoS_2_ under ns laser heating, and the energy coupling factors between OP and AP for the 71 nm thick MoSe_2_ under CW and ns lasers heating are also obtained. All the obtained coupling factors are listed in **Table** **3**. Figure 7d shows the distinguished temperatures of LO/TO phonon, ZO phonon, and acoustic phonon under ns laser heating for MoS_2_ (detailed in Section S1 in the Supporting Information). Similar to the CW case, LO/TO phonon has the highest temperature during laser heating. It can be seen that under ns laser heating, *G*_pp_|_ns_ of LO/TO phonon is much larger than that of ZO phonon. Consequently, the ZO phonon/AP temperature difference is much smaller than that of LO/TO phonon/AP. When comparing the contributions of Δ*T*_OA_ under CW and ns lasers, it can be observed that the contribution under CW laser is much larger than that under ns laser, as shown in Figure 7e,f. This is induced by two combined effects. First, under same laser intensity, ns laser heating case will have a lower overall temperature rise (Δ*T*_m_) since it does not reach steady state and ns laser case will have a lower Δ*T*_OA_ due to its higher *G*_pp_. Second, under the same laser intensity, Δ*T*_OA_ is reduced more than Δ*T*_m_ compared with the respective values of CW laser cases.

**Table 3:** Summary of *G*_pp_ for MoS_2_ and MoSe_2_

Laser	MoS_2_ (10^15^ W m^−3^ K^−1^]		MoSe_2_ (10^15^ W m^−3^ K^−1^]
	LO/TO phonon	ZO phonon	ZO phonon
CW	0.226	0.118	0.049
ns	2.277	0.424	0.193

For MoSe_2_, *κ*_∥_ obtained under 20× objective lens is 14.6 ± 0.6 W m^−1^ K^−1^ (detailed in Section S2 in the Supporting Information). Some previous studies have also measured *κ*_∥_ of MoSe_2_ of different thickness. For bulk MoSe_2_, *κ*_∥_ is about 35 W m^−1^ K^−1^.^[^ ^40^ ^]^ In our previous studies, we also measured *κ*_∥_ of MoSe_2_ with different thickness.^[^ ^11^ ^]^ There is an increasing trend for *κ*_∥_ with the increased thickness, mainly due to surface phonon scattering. The obtained *κ*_∥_ of 71 nm thick MoSe_2_ is reasonable and agrees well with that of similar thickness MoSe_2_ measured in our studies. Using the same process as for MoS_2_ case (detailed in Section S3 in the Supporting Information), we have obtained the ZO phonon and acoustic phonon temperature as shown in Figure 7g,h under CW and ns laser heating. The temperature difference between ZO phonon and AP decreases with the increased laser spot size. Also as quantified in Figure 7i, the percentage contribution of Δ*T*_OA_ is decreasing with the increased laser spot size. Figure 7i shows that the contribution of Δ*T*_OA_ under CW laser decreases from around 32% to around 6% when the laser spot size increases from 0.405 to 1.355 µm. While the contribution of Δ*T*_OA_ under ns laser decreases from around 20% to around 5% when the laser spot size increases from 0.311 to 1.060 µm. That is, the contribution of Δ*T*_OA_ under CW laser is larger than the contribution of Δ*T*_OA_ under ns laser.

Comparing with *G*_pp_ for MoS_2_, the corresponding *G*_pp_ for MoSe_2_ is much smaller. This reflects the relatively lower OP–AP coupling factor in MoSe_2_. Generally speaking, our results for MoS_2_ and MoSe_2_ all uncover weaker OP–AP energy coupling factors under CW laser heating than that under ns laser heating. This could be caused by the relatively higher electron population in ns laser heating and/or its stronger thermal nonequilibrium in space. Further studies are still needed to provide more detailed explanation in this area. Further discussion and explanation of *G*_pp_ are provided in the below section for graphene paper study.

### 2.5 AP–OP Energy Coupling Factor in Graphene Paper

Graphene has been relatively widely studied for its phonon behavior. In this section, we measure the energy coupling factor between LO/TO phonons and AP of GP and compare with first‐principles calculations to further the understanding. The in‐plane and crossplane thermal conductivities of GP used to determine the energy coupling factor have been measured with high confidence in our previous work.^[^ ^41^, ^42^ ^]^ The in‐plane thermal conductivity (*κ*_∥_) is measured using the transient electrothermal (TET) technique to be 634 W m^−1^ K^−1^ at room temperature. The crossplane thermal conductivity (*κ*_c_) is measured using a pulsed laser‐assisted thermal relaxation 2 (PLTR2) technique to be 6.08 W m^−1^ K^−1^ at room temperature.^[^ ^42^, ^43^ ^]^ More details of the measurement can be found in our previous work.^[^ ^42^ ^]^ All these thermal conductivity measurement involves negligible or does not involve electron–OP–AP nonequilibrium. Detailed discussions can be found in Section S4 in the Supporting Information.

Based on the measured *κ*_∥_ and *κ*_c_, steady state Raman experiment is conducted by using a CW laser. The G peak, which is associated with LO and TO phonon branches,^[^ ^44^ ^]^ is used in the experiment. Three objective lenses (20× , 50× , and 100× ) are used to obtain the corresponding *ψ*. A 3D heat conduction model is also used to obtain the AP temperature rise of GP under different laser spot sizes. The laser power used in the model is also very low (1 mW) to ensure a small temperature rise. The corresponding laser spot size measured in the experiment is also used in the modeling to guarantee the modeling accuracy. For the in‐plane thermal transport, the 2D kinetic equation has *κ*_∥_ = *Cv*_∥_ *l*_∥_/2, where *C* is volumetric heat capacity, *v*_∥_ (9171 m s^−1^) is the average phonon group velocity along the in‐plane direction, and *l*_∥_ is the phonon mean free path along the in‐plane direction. Based on our measured thermal conductivity, we have *l*_∥_ ≈ 142nm. In the crossplane direction, the phonon mean free path (*l*_c_) under room temperature has been studied in our previous work, and we have *l*_c_ ≈ 87 nm.^[^ ^42^ ^]^ As the radius of the laser spot in the experiments is about 300 nm or larger, the thermal transport can also be safely taken as diffusive and local equilibrium.

The assumption that Δ*T*_OA_ is proportional to the laser intensity is justified before the calculation of coupling factor for GP. An MTM developed by Ruan's group is used to calculate the temperature rise of different phonon branches and lattice in graphene.^[^ ^18^ ^]^ The governing equation for MTM is

(5) $$\begin{array}{c} \begin{array}{c} C_{e}\frac{\partial T_{e}}{\partial t}=\nabla \left(\kappa_{e}\nabla T_{e}\right)-\sum G_{\mathrm{ep},i}\left(T_{e}-T_{p,i}\right)+I/\tau e^{-z/\tau }\\ C_{p,i}\frac{\partial T_{p,i}}{\partial t}=\nabla \left(\kappa_{p}\nabla T_{p}\right)+G_{\mathrm{ep},i}\left(T_{e}-T_{p,i}\right)+G_{\mathrm{pp},i}\left(T_{\mathrm{Lattice}}-T_{p,i}\right) \end{array} \end{array}$$

where *i* is the index of phonon branches, and e and p refer to electron and phonon, respectively. *κ*, *C*, and *τ* refer to the energy carriers’ thermal conductivity and volumetric heat capacity, and optical absorption depth. *G*_pp,_ *_i_* is the coupling factor between each phonon branch and the lattice. All the room‐temperature values of input thermal properties, i.e., the thermal conductivity *κ*, the heat capacity *C*, the e*–*p coupling factor *G*_ep_, and the p*–*p coupling factor *G*_pp,_ *_i_*, used in the model can be found in Ruan's research.^[^ ^18^ ^]^ Then, by solving Equation (5) numerically, the temperature profiles of the different energy carriers are obtained. The absorbed laser power used in this modeling is still 1 mW, and the radius of the laser spot is 1.355 µm. Instead of simulating bulk GP, we use a 30‐nm thick GP suspended on a hole of 10 µm diameter. Such treatment is for convenience of modeling, and will not change the conclusion drawn below.

**Figure 8:**
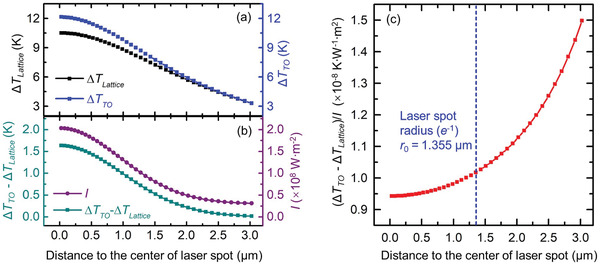
a) Temperature rise variation of TO phonons and lattice in space under CW laser irradiation. b) Variation of temperature rise difference between TO phonons and lattice in space. c) Ratio of temperature rise difference over laser intensity.

**Figure** **8**a shows the temperature rise distributions of TO phonons and lattice under laser irradiation. It can be seen that Δ*T*_TO_ is larger than Δ*T*_Lattice_, and the difference between them decreases with the increased distance from the center of laser spot. And, this difference variation is shown in Figure 8b. The laser intensity distribution is also shown in Figure 8b, which indicates that the variation of difference between Δ*T*_TO_ and Δ*T*_Lattice_ is similar to the laser intensity distribution in space. Figure 8c shows the (Δ*T*_TO_ − Δ*T*_Lattice_)/*I* distribution in space under the 20× objective lens. This value increases by only about 8% when the location moves from the laser spot center to the boundary of the laser spot. This firmly confirms that Δ*T*_OA_ can be treated proportional to *I*.

**Figure 9:**
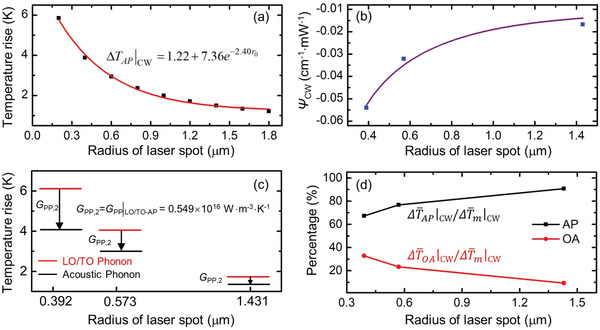
Data processing for determining contributions of Δ*T*_AP_ and Δ*T*_OA_ of GP under CW laser heating. a) Curve fitting for acoustic phonon temperature rise against laser spot size. b) Curve fitting for *ψ*_CW_ against laser spot size. c) Distinguished LO/TO phonon and AP temperatures under different laser spot sizes with 1 mW absorbed laser power. d) Contribution of Δ*T*_OA_ and Δ*T*_AP_ to the overall Δ*T*_m_.

As shown in **Figure** **9**a, the symbols are the data obtained based on the 3D heat conduction model of GP under 1 mW absorbed laser irradiation, the red line is the fitting curve. We have $\Delta {\bar{T}}_{\mathrm{AP}}{|}_{\mathrm{CW}}=1.22+7.36e^{-2.40r_{0}}$. It is a little different from that of MoS_2_ since the heat conduction in graphene paper is 3D. Then, the fitted relation between the temperature rise and laser spot size is used to fit the variation of *ψ* against laser spot size under CW laser. The *ψ*_CW_ values obtained under the three objective lenses and the fitting curve are shown in Figure 9b. Based on these fitting results, the coupling factor between LO/TO phonons and AP for GP is determined to be 0.549 × 10^16^ W m^−3^ K^−1^. This factor in fact is a value reflecting the LO/TO phonons coupling with all acoustic phonons. Ruan's group obtained the coupling factors between different phonon branches by developing a multitemperature model.^[^ ^18^, ^28^ ^]^ Based on their research, the average energy coupling factors for LO and TO phonon branches are 0.27 × 10^16^ and 0.14 × 10^16^ W m^−3^ K^−1^, respectively. Compared with these two values, it can be seen that the energy coupling factor obtained in our work is very close to the sum of two obtained values in their work (0.41 × 10^16^ W m^−3^ K^−1^). Figure 9c shows the distinguished LO/TO phonon and AP temperatures under different spot CW laser heating. The percentage contributions of Δ*T*_OA_ and Δ*T*_AP_ in the Raman measured temperature rise can also be obtained, shown in Figure 9d. As shown in Figure 9d, when the radius of laser spot is about 0.39 µm (under 100× lens), the contribution of Δ*T*_OA_ to the overall *ψ*_CW_ is about 34%. And this contribution decreases with the increased laser spot size. When the radius of laser spot is around 1.43 µm, the contribution of Δ*T*_OA_ is only around 9%.

The OP–AP energy coupling factor *G*_pp_ is related to the energy carrier relaxation time (*τ*_e_) as *G*_pp_ = *C*_p_/*τ*_e_.^[^ ^18^ ^]^ Here, *C*_p_ is the specific heat of phonons. For instance, for the G peak phonon branch in GP, *C*_p_ is the specific heat of LO and TO phonons. The observed difference in *G*_pp_ between MoS_2_ and MoSe_2_ and between different LO/TO and ZO branches listed in Table 3 is mainly attributed to the *C*_p_ difference and *τ*_e_ difference. Such observation has been clearly made and explained in the work by Lu et al.^[^ ^18^ ^]^ Take graphene as an example, *τ*_e_ is 388 ps for ZO phonon, and only 10 and 12 ps for LO and TO phonons. Although ZO phonon's specific heat (0.16 × 10^6^ J m^−3^ K^−1^) is higher than that of LO (0.03 × 10^6^ J m^−3^ K^−1^) and TO (0.02 × 10^6^ J m^−3^ K^−1^) phonons, but this will not offset the difference in their relaxation time. As a result, *G*_pp_ of ZO phonons is much smaller than that of LO and TO phonons.^[^ ^18^ ^]^ As for the observed increase of *G*_pp_ under ns laser heating over CW laser heating, it is speculated that the higher free electron population under ns laser heating could significantly intensify phonon scattering, reduce *τ*_e_, and thereby increase the OP–AP energy coupling factor. As pointed out by Lu et al.,^[^ ^18^ ^]^ during laser heating, ZO phonons tend to have much weaker interaction with electrons than LO and TO phonons. Therefore, the increase of electron population under ns laser heating will decrease the relaxation time of LO and TO phonons more significantly than that of ZO phonon. Consequently, compared with the CW case, in ns laser heating *G*_pp_ of LO/TO phonons will have much more increase than ZO phonons.

## 3 Conclusion

In this work, for the first time, we probed and distinguished the temperature rise of LO/TO, ZO, and acoustic phonons for 2D materials: MoS_2_, MoSe_2_, and graphene paper, and quantified the contribution on Δ*T*_OA_ in the Raman‐probed temperature rise. Under CW laser heating, Δ*T*_OA_ contribution can be more than 25% for MoS_2_, and >30% for MoSe_2_ and graphene paper. Such critical effects have never been considered in the widely reported Raman‐based thermal conductivity measurement of 2D materials. By excluding the OP–AP cascading energy transfer, we measured the intrinsic in‐plane thermal conductivity of 55 nm thick MoS_2_ and 71 nm thick MoSe_2_ as 46.9 ± 3.1 and 14.6 ± 0.6 W m^−1^ K^−1^, regardless of the Raman modes used in temperature probing. Also very critically, we characterized the energy coupling factor between OP and AP. *G*_pp_ is found in the order of 10^15^ W m^−3^ K^−1^ for MoS_2_ and 10^14^ W m^−3^ K^−1^ for MoSe_2_ under CW laser excitation. Under ns laser excitation, *G*_pp_ is found significantly increased, probably due to the more excited hot carriers. Still further study is needed in this area to look into the mechanisms. For GP, the characterized LO/TO phonon–AP coupling factor is 0.549 × 10^16^ W m^−3^ K^−1^, agreeing well with the first‐principles modeling result of 0.41 × 10^16^ W m^−3^ K^−1^.

## 4 Experimental Section

##### MoS_2_ and MoSe_2_ Preparation

The mechanical exfoliation method was used to prepare the nm‐thick MoS_2_ and MoSe_2_ sample with pristine, clean, and high‐quality structures.^[^ ^45^ ^]^ The sample preparation process is shown in Figure S1b–e in the Supporting Information. First, an adhesive Scotch tape was used to peel off a layered MoS_2_ or MoSe_2_ from corresponding bulk materials. Then, the layered MoS_2_ or MoSe_2_ was transferred to a gel film (Gel‐Film, PF‐20/1.5‐X4, Gel‐Pak). With the help of two 3D nanostages, the layered MoS_2_ or MoSe_2_ was transferred to the hole area on a silicon substrate.^[^ ^46^ ^]^ The hole was fabricated using the focused ion beam (FIB) technique and had a diameter of 10 µm. Both optical microscope and atomic force microscope (AFM) (NMAFM‐2, Digital Instruments, CA, USA) were used to identify and study the obtained suspended nm‐thick MoS_2_. Figure S1f in the Supporting Information shows the AFM image of the 55 nm thick MoS_2_. The thickness profile of the sample was measured along the red dashed line. The root‐mean‐square roughness (*R*_q_) value obtained from the dashed box area (5 µm × 5 µm) was used to evaluate the roughness (*R*_q_) of the sample. As shown in the figure, *R*_q_ value of the sample was 2.85 nm. Compared with the thickness of the sample, this value was relatively small.

##### Structure Characterization of GP

GP was purchased from Graphene Supermarket. X‐ray diffraction (XRD) and X‐ray photoelectron spectroscopy (XPS) had been used to study the purity level and elemental composition of GP in the previous work. A sharp and distinct peak around 26.6° was observed in XRD characterization, corresponding to the (002) peak. The interlayer spacing of GP was determined to be 3.35 Å, which was the same as pristine natural graphite. This result indicated that the GP had excellent ordered structure. XPS was used to do the chemical analysis. The elemental composition was determined as: C 1s (98.91%), O 1s (0.66%), and F 1s (0.43%), which also indicated that the GP was composed of highly purified graphene flakes. Based on the previous work, the in‐plane structure domain size of GP was around 1.68 µm, and the crossplane structure domain size of GP was around 375 nm.^[^ ^41^, ^42^ ^]^

## Conflict of Interest

The authors declare no conflict of interest.

## Supporting information

Supporting Information

## References

[1] H. Malekpour , A. A. Balandin , J. Raman Spectrosc. 2018, 49, 106.

[2] C. Ferrante , A. Virga , L. Benfatto , M. Martinati , D. De Fazio , U. Sassi , C. Fasolato , A. K. Ott , P. Postorino , D. Yoon , G. Cerullo , F. Mauri , A. C. Ferrari , T. Scopigno , Nat. Commun. 2018, 9, 308. 29358728 10.1038/s41467-017-02508-x PMC5778087

[3] X. Zhang , D. Sun , Y. Li , G. H. Lee , X. Cui , D. Chenet , Y. You , T. F. Heinz , J. C. Hone , ACS Appl. Mater. Interfaces 2015, 7, 25923. 26517143 10.1021/acsami.5b08580

[4] J. J. Bae , H. Y. Jeong , G. H. Han , J. Kim , H. Kim , M. S. Kim , B. H. Moon , S. C. Lim , Y. H. Lee , Nanoscale 2017, 9, 2541. 28150838 10.1039/c6nr09484h

[5] R. Yan , J. R. Simpson , S. Bertolazzi , J. Brivio , M. Watson , X. Wu , A. Kis , T. Luo , A. R. Hight Walker , H. G. Xing , ACS Nano 2014, 8, 986. 24377295 10.1021/nn405826k

[6] X. Li , J. Zhang , A. A. Puretzky , A. Yoshimura , X. Sang , Q. Cui , Y. Li , L. Liang , A. W. Ghosh , H. Zhao , R. R. Unocic , V. Meunier , C. M. Rouleau , B. G. Sumpter , D. B. Geohegan , K. Xiao , ACS Nano 2019, 13, 2481. 30673215 10.1021/acsnano.8b09448

[7] J. S. Reparaz , E. Chavez‐Angel , M. R. Wagner , B. Graczykowski , J. Gomis‐Bresco , F. Alzina , C. M. Sotomayor Torres , Rev. Sci. Instrum. 2014, 85, 034901. 24689609 10.1063/1.4867166

[8] Q.‐Y. Li , X. Zhang , K. Takahashi , Int. J. Heat Mass Transfer 2018, 125, 1230.

[9] S. Xu , T. Wang , D. Hurley , Y. Yue , X. Wang , Opt. Express 2015, 23, 10040. 25969045 10.1364/OE.23.010040

[10] T. Wang , M. Han , R. Wang , P. Yuan , S. Xu , X. Wang , J. Appl. Phys. 2018, 123, 145104.

[11] H. Zobeiri , R. Wang , T. Wang , H. Lin , C. Deng , X. Wang , Int. J. Heat Mass Transfer 2019, 133, 1074.

[12] P. Yuan , R. Wang , T. Wang , X. Wang , Y. Xie , Phys. Chem. Chem. Phys. 2018, 20, 25752. 30283921 10.1039/c8cp02858c

[13] P. Yuan , R. Wang , H. Tan , T. Wang , X. Wang , ACS Photonics 2017, 4, 3115.

[14] P. Yuan , H. Tan , R. Wang , T. Wang , X. Wang , RSC Adv. 2018, 8, 12767. 10.1039/c8ra01106k PMC9079430 35541278

[15] R. Wang , T. Wang , H. Zobeiri , P. Yuan , C. Deng , Y. Yue , S. Xu , X. Wang , Nanoscale 2018, 10, 23087. 30511715 10.1039/c8nr05641b

[16] H. Zobeiri , R. Wang , Q. Zhang , G. Zhu , X. Wang , Acta Mater. 2019, 175, 222.

[17] S. Sullivan , A. Vallabhaneni , I. Kholmanov , X. Ruan , J. Murthy , L. Shi , Nano Lett. 2017, 17, 2049. 28218545 10.1021/acs.nanolett.7b00110

[18] Z. Lu , A. Vallabhaneni , B. Cao , X. Ruan , Phys. Rev. B 2018, 98, 134309.

[19] L. Waldecker , R. Bertoni , R. Ernstorfer , J. Vorberger , Phys. Rev. X 2016, 6, 021003.

[20] Z. Tian , K. Esfarjani , J. Shiomi , A. S. Henry , G. Chen , Appl. Phys. Lett. 2011, 99, 053122.

[21] E. Pop , R. W. Dutton , K. E. Goodson , J. Appl. Phys. 2004, 96, 4998.

[22] A. Mittal , S. Mazumder , J. Heat Transfer 2010, 132, 052402.

[23] L. Lindsay , D. A. Broido , N. Mingo , Phys. Rev. B 2010, 82, 115427.

[24] B. P. Falcão , J. P. Leitão , M. R. Correia , M. R. Soares , H. Wiggers , A. Cantarero , R. N. Pereira , Phys. Rev. B 2017, 95, 115439.

[25] R. A. Escobar , C. H. Amon , J. Heat Transfer 2007, 129, 790.

[26] L. Chen , S. Kumar , J. Appl. Phys. 2012, 112, 043502.

[27] Z. Wei , J. Yang , K. Bi , Y. Chen , J. Appl. Phys. 2014, 116, 153503.

[28] A. K. Vallabhaneni , D. Singh , H. Bao , J. Murthy , X. Ruan , Phys. Rev. B 2016, 93, 125432.

[29] I. Chatzakis , H. Yan , D. Song , S. Berciaud , T. F. Heinz , Phys. Rev. B 2011, 83, 205411.

[30] P. G. Klemens , Phys. Rev. 1966, 148, 845.

[31] C. Ulrich , E. Anastassakis , K. Syassen , A. Debernardi , M. Cardona , Phys. Rev. Lett. 1997, 78, 1283.

[32] Y. Cai , J. Lan , G. Zhang , Y.‐W. Zhang , Phys. Rev. B 2014, 89, 035438.

[33] A. Molina‐Sánchez , L. Wirtz , Phys. Rev. B 2011, 84, 155413.

[34] A. Taube , J. Judek , A. Lapinska , M. Zdrojek , ACS Appl. Mater. Interfaces 2015, 7, 5061. 25706435 10.1021/acsami.5b00690

[35] X. Wei , Y. Wang , Y. Shen , G. Xie , H. Xiao , J. Zhong , G. Zhang , Appl. Phys. Lett. 2014, 105, 103902.

[36] J. Liu , G.‐M. Choi , D. G. Cahill , J. Appl. Phys. 2014, 116, 233107.

[37] I. Jo , M. T. Pettes , E. Ou , W. Wu , L. Shi , Appl. Phys. Lett. 2014, 104, 201902.

[38] C. Yim , M. O'Brien , N. McEvoy , S. Winters , I. Mirza , J. G. Lunney , G. S. Duesberg , Appl. Phys. Lett. 2014, 104, 103114.

[39] P. Yuan , C. Li , S. Xu , J. Liu , X. Wang , Acta Mater. 2017, 122, 152.

[40] P. Jiang , X. Qian , X. Gu , R. Yang , Adv. Mater. 2017, 29, 1701068. 10.1002/adma.201701068 28727182

[41] Y. Xie , P. Yuan , T. Wang , N. Hashemi , X. Wang , Nanoscale 2016, 8, 17581. 27714159 10.1039/c6nr06402g

[42] M. Han , J. Liu , Y. Xie , X. Wang , Carbon 2018, 126, 532.

[43] M. Han , Y. Xie , J. Liu , J. Zhang , X. Wang , Nanotechnology 2018, 29, 265702. 29620536 10.1088/1361-6528/aabbc9

[44] J. B. Wu , M. L. Lin , X. Cong , H. N. Liu , P. H. Tan , Chem. Soc. Rev. 2018, 47, 1822. 29368764 10.1039/c6cs00915h

[45] H. Li , J. Wu , Z. Yin , H. Zhang , Acc. Chem. Res. 2014, 47, 1067. 24697842 10.1021/ar4002312

[46] A. Castellanos‐Gomez , M. Buscema , R. Molenaar , V. Singh , L. Janssen , H. S. J. van der Zant , G. A. Steele , 2D Mater. 2014, 1, 011002.

