# Supplementary material for: Distinguishing Optical and Acoustic Phonon Temperatures and Their Energy Coupling Factor under Photon Excitation in nm 2D Materials
Source: Adv Sci (Weinh). 2020 May 26;7(13):2000097. doi: 10.1002/advs.202000097 (PMC7341092; doi:10.1002/advs.202000097)
Supplement: Supplementary file 1 — Supporting Information [file ADVS-7-2000097-s001.pdf]

Supporting Information

Distinguish Optical and Acoustic Phonon Temperatures and Their Energy Coupling Factor under Photon Excitation in nm 2D Materials

Ridong Wang [1], Hamidreza Zobeiri [1], Yangsu Xie, Xinwei Wang [*], Xing Zhang [*], Yanan Yue [*]

Prof. R. Wang
State Key Laboratory of Precision Measuring Technology and Instruments, Tianjin University, Tianjin 300072, P. R. China.

H. Zobeiri, Prof. X. Wang
Department of Mechanical Engineering, Iowa State University, Ames, Iowa 50011, United States.
E-mail: xwang3@iastate.edu

Prof. Y. Xie
College of Chemistry and Environmental Engineering, Shenzhen University, Shenzhen, Guangdong 518055, P. R. China.

Prof. X. Zhang
Key Laboratory for Thermal Science and Power Engineering of Ministry of Education, Department of Engineering Mechanics, Tsinghua University, Beijing 100084, P. R. China.
E-mail: x-zhang@mail.tsinghua.edu.cn

Prof. Y. Yue
School of Power and Mechanical Engineering, Wuhan University, Wuhan 430072, P. R. China.
E-mail: yyue@whu.edu.cn

[1] These authors contributed equally to this work.

S1. Distinguish Temperatures of AP and OP and Determine Energy Coupling Factor between Them for 55 nm-thick MoS_2 under ns laser heating

For the ns laser, the laser intensity is given as:

$$I_{\mathrm{ns}} = \left(I_0 / \tau_L\right)\exp\left(-r^2/r_0^2\right)\exp\left(-z/\tau_L\right)\exp\left[-t^2/t_0^2\right], \tag{S1}$$

where $I_0 = P/(f\cdot\sqrt{\pi}\cdot t_0\cdot\pi r_0^2)$ is the laser power per unit area at the center of laser spot, f (300 KHz) is the repetition rate of ns laser, t_0 (127 ns) is the characteristic time of ns laser. Based on Equation (1) and (S1), the Raman intensity weighted temperature rise under ns laser case can be written as:

$$\overline{\Delta T}_m\Big|_{\mathrm{ns}} = \frac{\iiint \Delta T_m\big|_{\mathrm{ns}} I_{\mathrm{ns}} e^{-z/\tau} 2\pi r dr dz dt}{\iiint I_{\mathrm{ns}} e^{-z/\tau} 2\pi r dr dz dt} = \overline{\Delta T}_{AP}\Big|_{\mathrm{ns}} + \frac{1}{4\sqrt{2}}\cdot\frac{I_0}{\tau_L}\cdot\frac{\delta}{G_{\mathrm{pp}}\big|_{\mathrm{ns}}}. \tag{S2}$$

Then the relation between ψ_{ns} and $\overline{\Delta T}_m\big|_{\mathrm{ns}}$ can be written as:

$$\psi_{\mathrm{ns}} = \mathrm{A}\cdot\left[(5.76+28.07e^{-1.88r_0}) + \frac{1}{4\sqrt{2}}\cdot\frac{P}{f\cdot\sqrt{\pi}\cdot t_0\cdot\pi r_0^2\cdot\tau_L}\cdot\frac{\delta}{G_{\mathrm{pp}}\big|_{\mathrm{ns}}}\right]\Big/P\ . \tag{S3}$$

Here, δ is also taken as 2/3 for E^1_{2g} mode, and 1/3 for A_{1g} mode. Similarly, ψ_{ns} is also proportional to the corresponding temperature rise. That is, ψ_{ns} can be used to obtain $G_{\mathrm{pp}}\big|_{\mathrm{ns}}$. Then a 3D numerical modeling method based on the finite volume method is conducted to calculate the temperature rise under the ns laser with different laser spot size. Figure S4(a) shows the variation of $\overline{\Delta T}_{AP}\big|_{\mathrm{ns}}$ against laser spot size in our modeling for the 55 nm-thick MoS_2. An exponential fitting method is used to fit the data to develop the relation between the laser spot size and temperature rise under ns laser heating. The relation is also shown in Figure S4(a) (for 20 µW absorbed laser power). Comparing with the exponential power under CW laser, the exponential power under ns laser is larger. Under CW laser, the heat conduction spreads out the temperature, so the laser spot size effect is relatively weak. Under ns laser, the temperature of the sample does not reach steady state. Therefore, the temperature distribution

is more tightly related to the laser spot size.

Then, the fitted relation above is used to fit the variation of ψ_{ns} values against laser spot size based on Equation (S2) and (S3). The ψ_{ns} values obtained under the three objective lenses and the fitting curves are shown in Figure S4(b) for the two Raman modes. Based on these fitting results, the energy coupling factors between OP and AP for the two Raman modes under transient state are obtained: 2.277×10^{15} $W\cdot m^{-3}\cdot K^{-1}$ for E^1_{2g} mode and 0.424×10^{15} $W\cdot m^{-3}\cdot K^{-1}$ for A_{1g} mode. $G_{pp}\big|_{ns}$ for E^1_{2g} mode is much larger than $G_{pp}\big|_{ns}$ for A_{1g} mode. In addition, $G_{pp}\big|_{ns}$ is larger than $G_{pp}\big|_{CW}$ for both modes. Based on the fitting results from Figure S4(b), the contribution of ΔT_{OA} and ΔT_{AP} to the measured ΔT_m can be distinguished from the Raman results. The distinguished temperatures of LO/TO phonon, ZO phonon, and AP are shown in Figure 7(d).

S2. Intrinsic thermal conductivity determination of $MoSe_2$

Figure S5(a) – (c) shows the 2D contour maps of the Raman peak of $MoSe_2$ under CW laser with 20×, 50×, and 100× objective lens, respectively. The peak is also observed to redshift with the increased laser power. Five representative room temperature Raman spectra under CW laser with the three objective lenses are shown in Figure S5(d) – (f). The results also confirm that the Raman peak are redshifted with the increased laser power. As shown in Figure S5(g) – (i), there is also a good linear relationship between the position of the peak and the laser power. Similar observations are also made under ns laser as shown in Figure S6. The obtained ψ values also decrease with the increased laser spot size for both CW and ns laser heating.

3D numerical modeling is conducted to determine the in-plane thermal conductivity of $MoSe_2$ with

different objective lenses. Figure S7(a) shows that the κ_{eff} based on the Raman peak can be obtained by interpolating the experimental results in the theoretical curves. All the obtained κ_{eff} values are summarized in Figure S7(b). As shown in this figure, the κ_{eff} obtained based on the A_{1g} mode decreases from 11.5±0.9 $W \cdot m^{-1} \cdot K^{-1}$ to 7.0±0.4 $W \cdot m^{-1} \cdot K^{-1}$ when the objective lens changes from 100× to 50×, then increases to 14.6±0.6 $W \cdot m^{-1} \cdot K^{-1}$ when the objective lens changes from 50× to 20×. This phenomenon also exists for the A_{1g} mode of MoS_2. This phenomenon could be due to the following reasons: (1) the laser spot size difference between CW and ns lasers under the same objective lens, (2) the G_{pp} difference between CW and ns laser heating cases.

S3. Distinguish Temperatures of AP and OP and Determine Energy Coupling Factor between Them for $MoSe_2$

The coupling factors between OP and AP for the 71 nm-thick $MoSe_2$ under CW and ns lasers can also be obtained based on Figure S4(c) and (d). For $MoSe_2$, only A_{1g} mode is detected from the Raman spectra. The κ_{eff} under the 20× objective lens is used to calculate the coupling factors. The obtained coupling factors under CW and ns laser are 0.049×10^{15} $W \cdot m^{-3} \cdot K^{-1}$ and 0.193×10^{15} $W \cdot m^{-3} \cdot K^{-1}$, respectively.

S4. In-plane and out-of-plane Thermal Conductivities of Graphene Paper

The in-plane and cross-plane thermal conductivities of graphene paper (GP) have been measured with high confidence in our previous work.[1, 2] The in-plane thermal conductivity ($\kappa_{\parallel}$) is measured using the transient electrothermal (TET) technique, in which a small step DC current is applied to induce Joule heating in a suspended GP sample.[3, 4] The temperature of GP first increases and then becomes stable through heat conduction and thermal radiation. The voltage evolution during this process is measured

to probe the temperature variation. The normalized average temperature rise which is defined as $T^*(t) = [T(t) - T_0] \big/ [T(t \to \infty) - T_0]$, where $T(t \to \infty)$ is the temperature at steady state, can be written as below:

$$T^* = \frac{48}{\pi^4} \sum_{m=1}^{\infty} \frac{1-(-1)^m}{m^2} \frac{1-\exp\left[-m^2\pi^2\alpha_{eff} t / L^2\right]}{m^2}. \tag{S4}$$

Here α_{eff} is the effective thermal diffusivity including the radiation effect. The real thermal diffusivity α can be obtained by subtracting the contribution of radiation as $\alpha = \alpha_{eff} - 8\varepsilon\sigma \overline{T}^3 L^2 / \pi^2 D \rho c_p$, where ρc_p is the volumetric specific heat, ε is the emissivity, σ is the Stefan-Boltzmann constant, $\overline{T}$ is the average temperature of the sample during the heating process, L is the sample length, and D is its thickness. Then, we can calculate $\kappa_{\parallel}$ as $\alpha \cdot \rho c_p$. The obtained $\kappa_{\parallel}$ of GP is 634 $W \cdot m^{-1} \cdot K^{-1}$ at room temperature.

In TET, we fit the trend of the temperature change against time to determine the thermal diffusivity, then determine the thermal conductivity. The electrons-OP and OP-AP temperature difference will only add a constant value on the AP temperature, and does not affect the fitting results. Note in the TET technique, since the heating is over the whole sample and the heat current is significantly lower than the laser intensity in this work, the electron temperature is very close to that of the AP and their temperature difference is negligible compared with the measured temperature rise. In the TET technique, the temperature rise of the sample is obtained by $\Delta T = I^2 RL / 12 A_c \kappa_{\parallel}$. In the equation, I is the current flowing through the sample, R is its resistance at steady state, and A_c is its cross-sectional area. During the experiment, as the applied DC current is very small, the induced temperature rise of the sample is also very small (0.5~1.5 K). Here we take an extreme temperature rise of 2 K in TET measurement for analysis. Under such Joule heating, the temperature difference between electron and

AP can be calculated by $T_e - T_{AP} = I^2 R V^{-1}\left[\left(\sum G_{\mathrm{ep}}\right)^{-1} + \left(\sum G_{\mathrm{pp}}\right)^{-1}\right]$, where V is the volume of the sample, G_{ep} is the coupling factor between electrons and OP, G_{pp} is the coupling factor between OP and AP. As there are three phonon branches for both OP and AP, here the sums of all the corresponding coupling factors are used in the calculation. Based on the coupling factors obtained by Ruan's group, $\sum G_{\mathrm{ep}}$ and $\sum G_{\mathrm{pp}}$ are 0.33×10^{16} and 0.45×10^{16} W·m^{-3}·K^{-1}, respectively.[5] Then, the temperature difference between electrons and AP is calculated to be around 2.8×10^{-8} K, which is negligible compared with the measured temperature rise of 2 K.

The cross-plane thermal conductivity (κ_c) is measured using a pulsed laser-assisted thermal relaxation 2 (PLTR2) technique.[2, 6] In this technique, the sample is heated at the front side using a nanosecond laser of 8 ns pulse width. Then, the temperature of the Ir coating on the other side of the sample increases to a maximum value. A DC current is applied to the Ir coating (which is insulated from GP by nm-thick PMMA) to monitor the electrical resistance, which can be used to probe the temperature variation at the backside of the sample. As the laser pulse width is very short, the sample does not experience laser heating all the time and the AP and OP quickly reach the same temperature after the laser heating. As a result, there is nearly no temperature difference between AP and OP after laser heating. By using the PLTR2 technique, κ_c of GP is determined as 6.08 W·m^{-1}·K^{-1} at room temperature. More details of the measurement can be found in our previous work.[2] All these intrinsic thermal conductivity data of GP are used in the below data processing.

For GP, ns ET-Raman technique is not used for direct thermal conductivity measurement. As GP has a very high thermal conductivity, an extremely high laser power density is needed to induce a measurable temperature rise. However, unusual broadening of the D band and splitting of the G band into G$^-$ and

G^+ components was observed during long-term laser exposure at power densities higher than 1600 kW·cm^{-2}.[7] For CW laser case, the maximum laser power density used is around 1300 kW·cm^{-2}, which is close to the damage threshold. For ns laser case, the maximum laser power is significantly higher than 1600 kW·cm^{-2} in order to generate a measurable temperature rise. Figure S8 shows the Raman spectra of GP under CW and ns lasers with a 100× objective lens. In addition to the G and 2D peaks under both lasers, a peak around 1940 cm^{-1} is identified only under ns laser. This peak is called iTALO$^+$ mode, which is a combination of out-of-plane TO and LO phonons around the K point of the graphene Brillouin zone.[8] This mode appears for single layer and AB-stacked graphene, which indicates that the structure of GP is changed under the ns laser. As a result, $\kappa_{\parallel}$ and κ_c measured by the TET and PLTR2 technique are used for our data processing. And these two values already have negligible effect from the temperature difference between OP and AP.

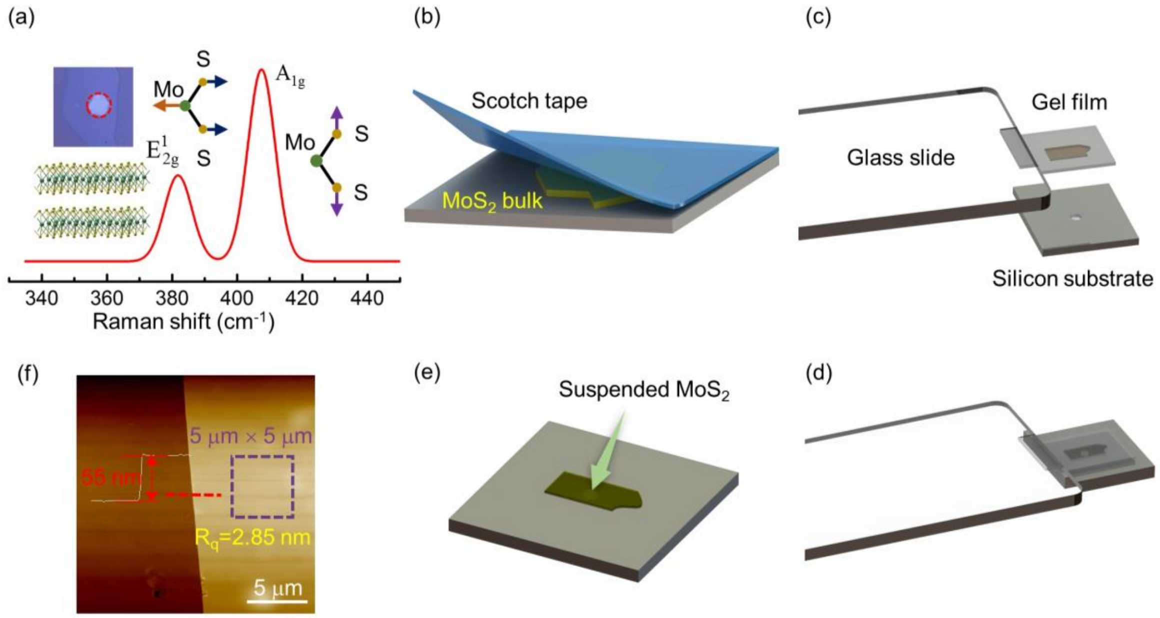

Figure S1. Sample preparation and identification of the MoS_2 nanosheet. (a) Representative Raman spectra of the suspended MoS_2 sample. The top left inset shows the optical image of the suspended sample. The other left inset shows the atomic structure of MoS_2. The distance between two adjacent layers is about 0.65 nm. (b) nm-thick MoS_2 nanosheet is mechanically exfoliated from the bulk by using a Scotch tape. (c) The exfoliated nanosheet is then transferred from the Scotch tape to a gel film. The gel film is attached to the edge of a glass slide and aligned with the hole in the silicon substrate by using two 3D nano-stages. (d) The gel film is moved down to touch the silicon substrate after alignment. (e) With moving up the gel film slowly, the MoS_2 or $MoSe_2$ nanosheet is transferred to the silicon substrate. (f) AFM image of 55 nm thick MoS_2 suspended on silicon substrate. The dashed box is an area used to evaluate the roughness of the sample. The thickness profile of the sample is measured along the red dashed line in the image.

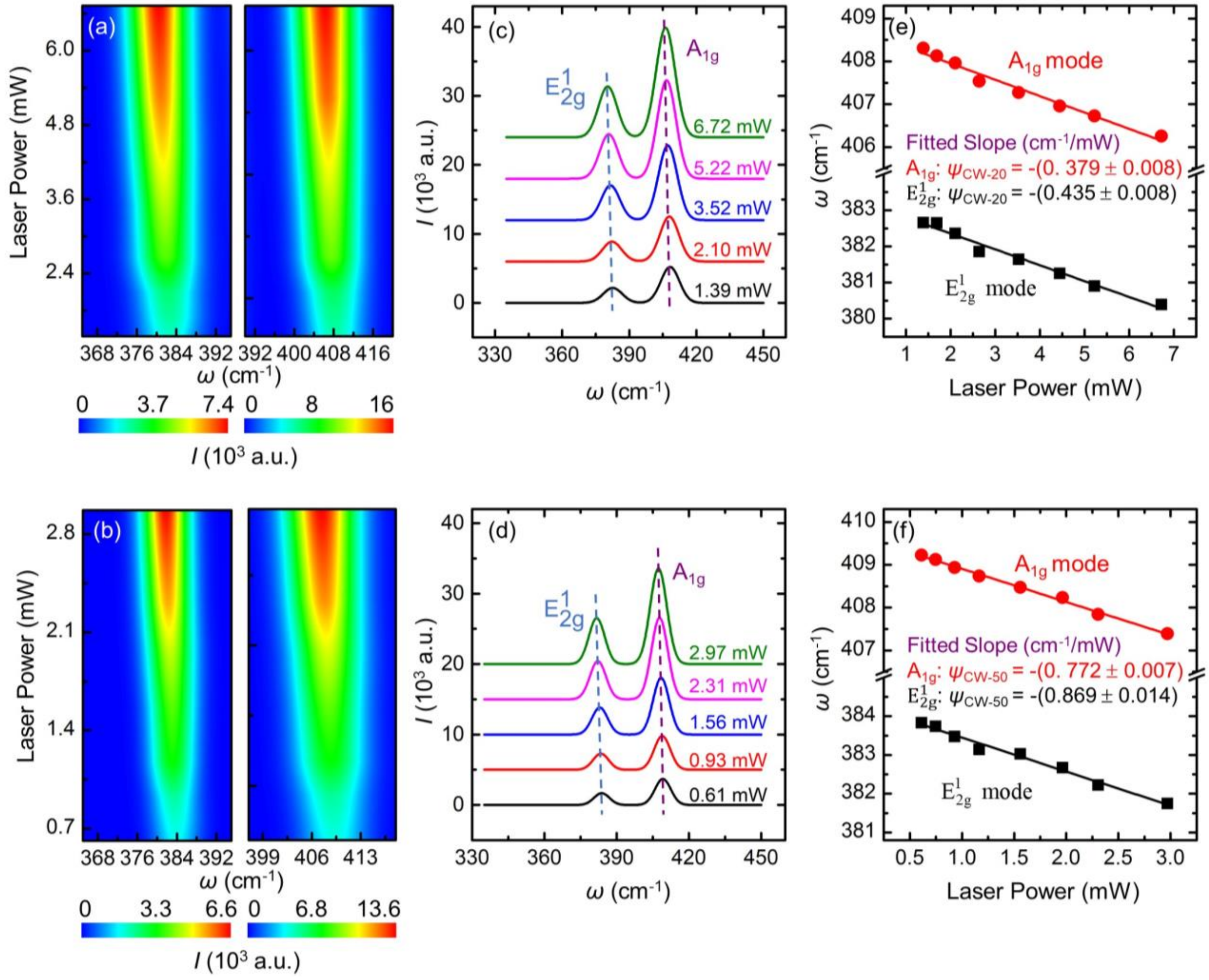

Figure S2. (a)-(b) 2D contour maps of MoS_2 Raman peaks. These three figures demonstrate the variation of Raman shift against the laser power of CW laser with 20× and 50× objective lenses, respectively. (c)-(d) Five representative Raman spectra of MoS_2 with increased laser power of CW laser with 20× and 50× objective lens, respectively. Both modes are redshifted with increased laser power. For CW laser with the two objective lenses, the Raman shifts of the two modes as a function of laser power are shown in (e) and (f).

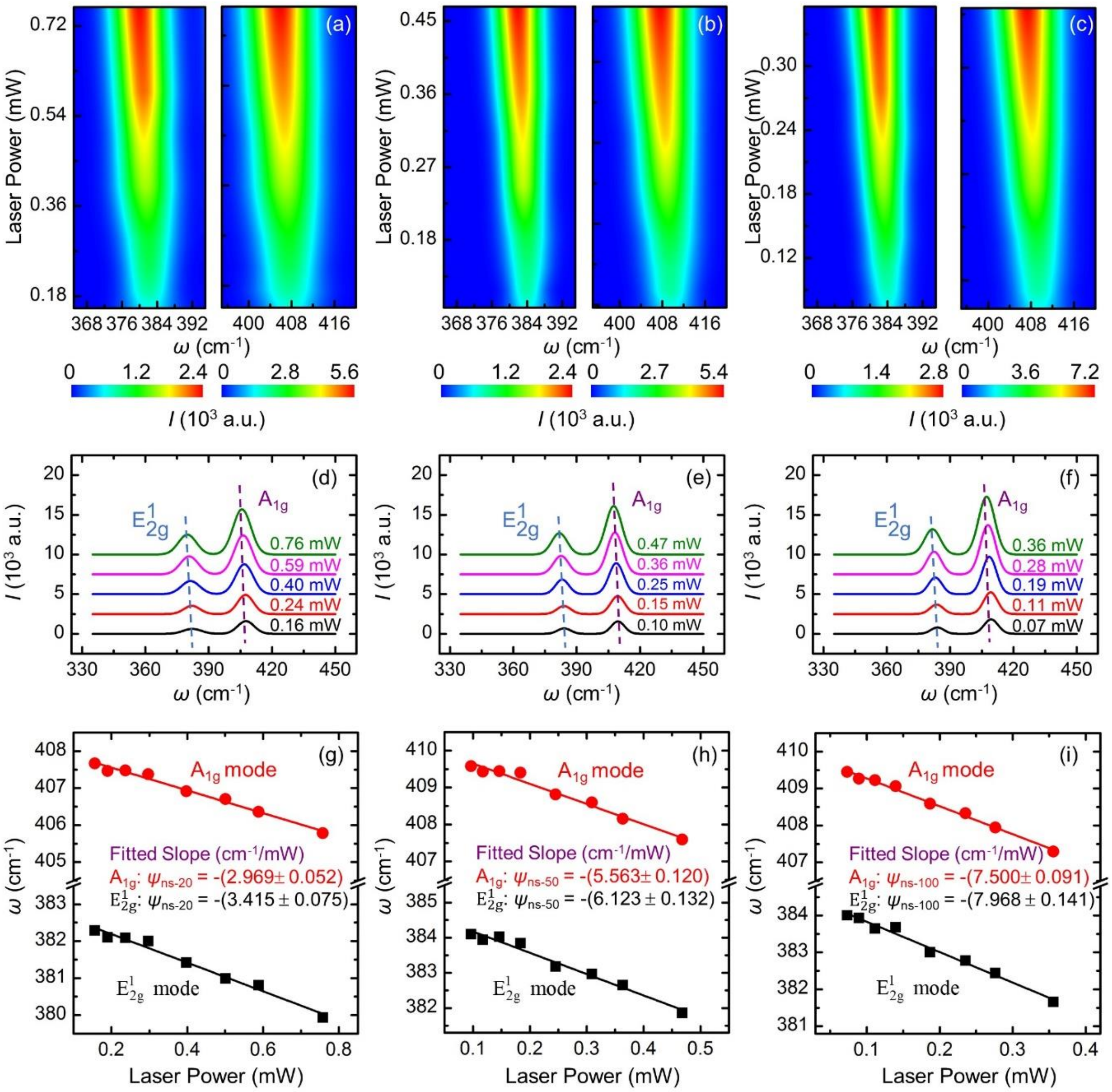

Figure S3. (a)-(c) 2D contour maps of MoS_2 Raman peaks. These three figures demonstrate the variation of Raman shift against the laser power of ns laser with 20×, 50×, and 100× objective lenses, respectively. (d)-(f) Five representative Raman spectra of MoS_2 with increased laser power of ns laser with 20×, 50×, and 100× objective lens, respectively. Both modes are redshifted with increased laser power. For ns laser with the three objective lenses, the Raman shifts of the two modes as a function of laser power are shown in (g-i).

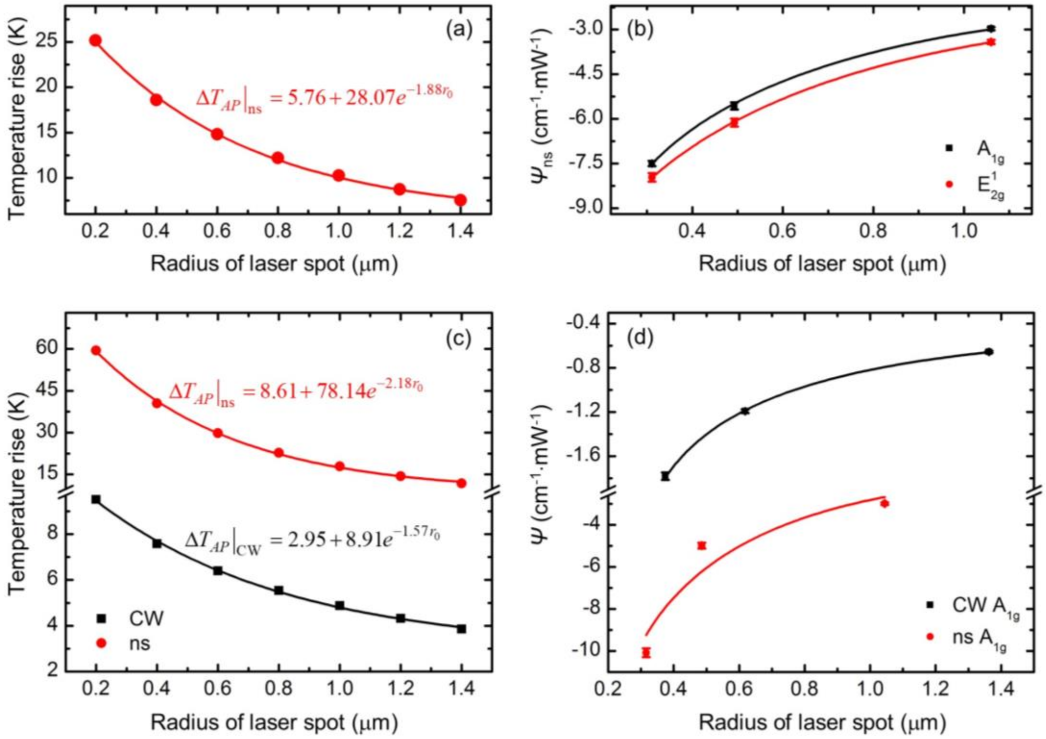

Figure S4. (a) Theoretical temperature rise against laser spot size under ns laser for MoS_2. (b) ψ against laser spot size curve fitting under ns laser heating for MoS_2. (c) Theoretical temperature rise against laser spot size under CW and ns lasers for $MoSe_2$. (d) ψ against laser spot size curve fitting under CW and ns laser heating for $MoSe_2$.

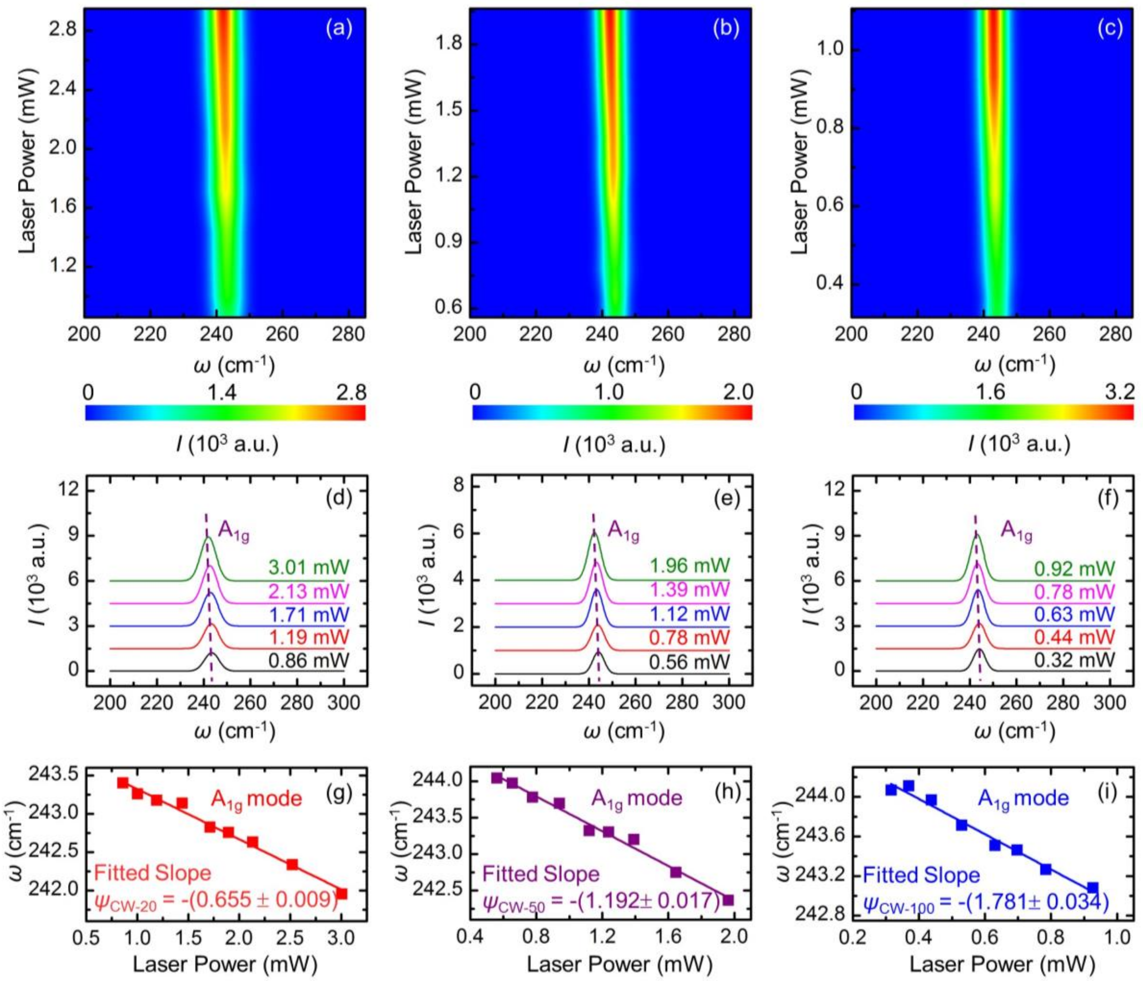

Figure S5. (a)-(c) 2D contour maps of $MoSe_2$ Raman peak. These three figures demonstrate the variation of Raman shift against the laser power of CW laser with 20×, 50×, and 100× objective lenses, respectively. (d)-(f) Five representative Raman spectra of $MoSe_2$ with increased laser power of CW laser with 20×, 50×, and 100× objective lenses, respectively. The Raman peak is redshifted with increased laser power. For the CW laser with the three objective lenses, the Raman shift of A_{1g} mode as a function of laser power are shown in (g-i).

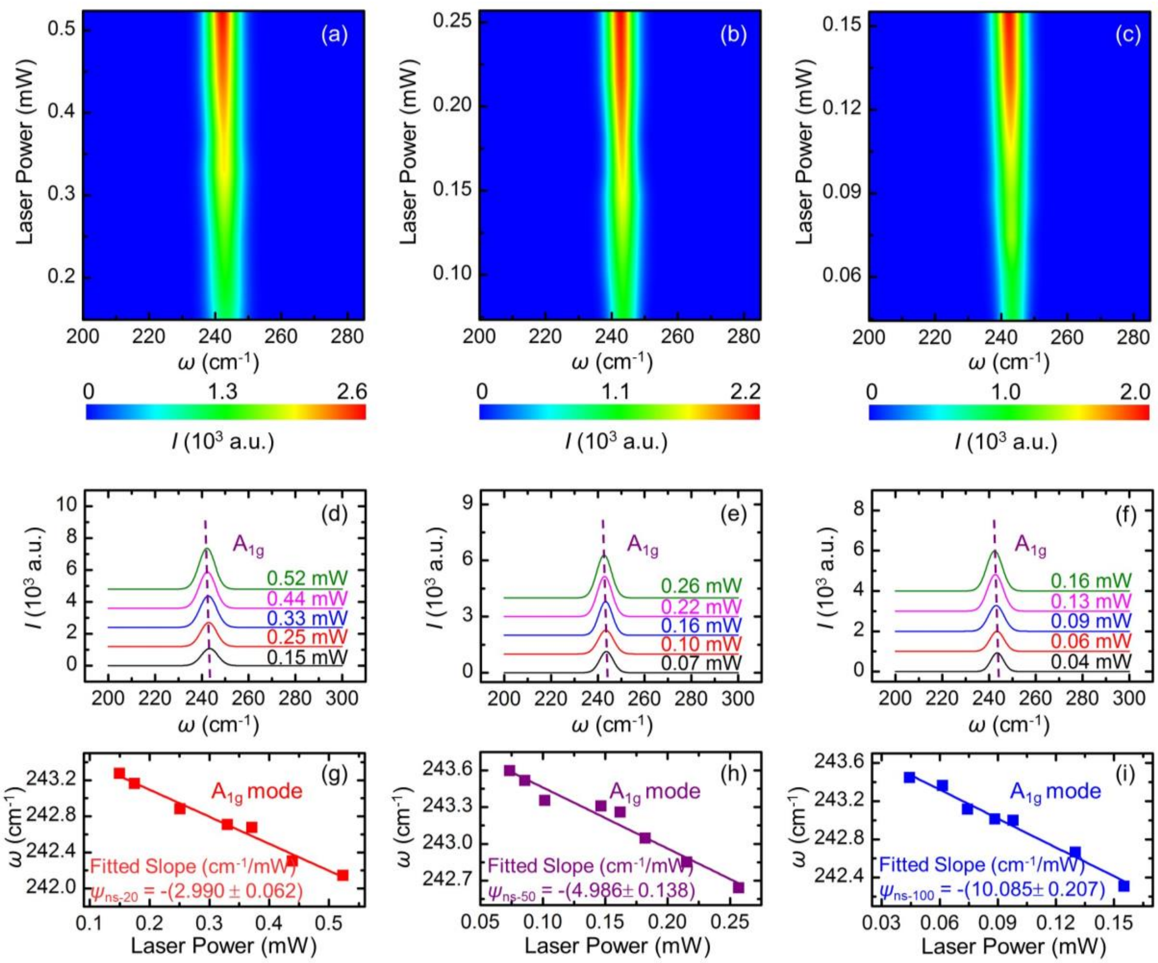

Figure S6. (a)-(c) 2D contour maps of $MoSe_2$ Raman peak. These three figures demonstrate the variation of Raman shift against the laser power of ns laser with 20×, 50×, and 100× objective lenses, respectively. (d)-(f) Five representative Raman spectra of $MoSe_2$ with increased laser power of ns laser with 20×, 50×, and 100× objective lenses, respectively. The Raman peak is redshifted with increased laser power. For ns laser with the three objective lenses, the Raman shift of A_{1g} mode as a function of laser power are shown in (g-i).

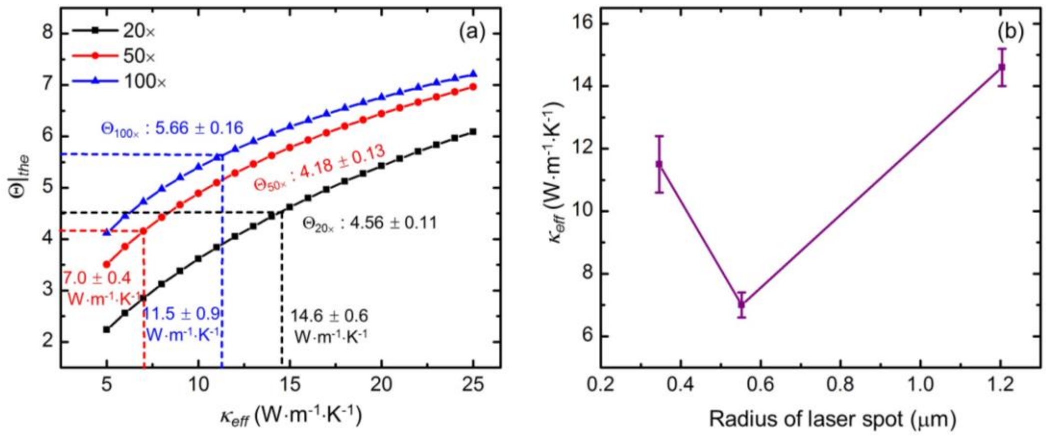

Figure S7. 3D numerical modeling and data processing results for the 71 nm-thick $MoSe_2$. The in-plane κ_{eff} is obtained by interpolating the experimental results in the curves. (a) In-plane κ_{eff} obtained under the 20×, 50×, and 100× objective lenses for the A_{1g} Raman mode. (b) In-plane κ_{eff} variation against laser spot size of the A_{1g} Raman mode.

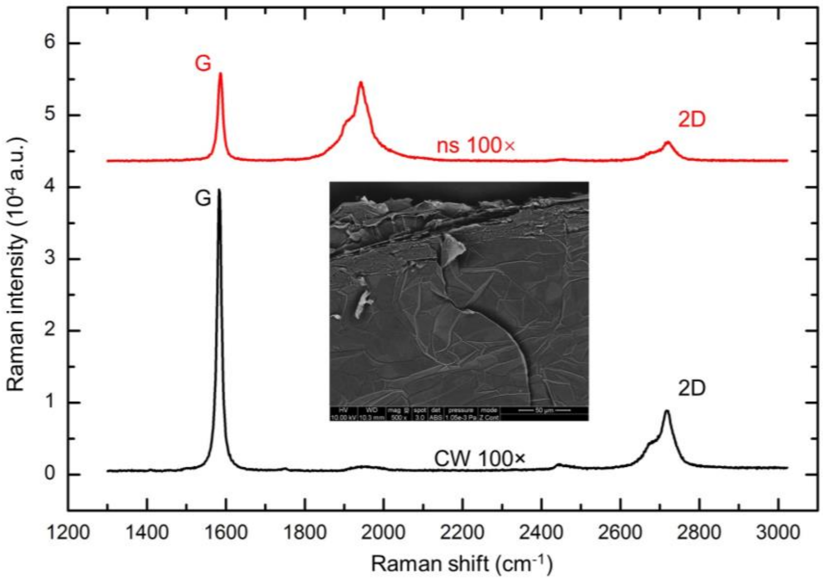

Figure S8. Raman spectra of graphene paper under CW and ns lasers with a 100× objective lens. The inset image shows an SEM image under 100× magnification.

References

[1] Y. Xie, P. Yuan, T. Wang, N. Hashemi, X. Wang, *Nanoscale* **2016**, 8, 17581.
[2] M. Han, J. Liu, Y. Xie, X. Wang, *Carbon* **2018**, 126, 532.
[3] J. Liu, W. Qu, Y. Xie, B. Zhu, T. Wang, X. Bai, X. Wang, *Carbon* **2017**, 121, 35.
[4] R. Wang, H. Zobeiri, H. Lin, W. Qu, X. Bai, C. Deng, X. Wang, *Carbon* **2019**, 147, 58.
[5] Z. Lu, A. Vallabhaneni, B. Cao, X. Ruan, *Phys Rev B* **2018**, 98, 134309.
[6] M. Han, Y. Xie, J. Liu, J. Zhang, X. Wang, *Nanotechnology* **2018**, 29, 265702.
[7] Y. Stubrov, A. Nikolenko, V. Strelchuk, S. Nedilko, V. Chornii, *Nanoscale Research Letters* **2017**, 12, 297.
[8]R. Rao, R. Podila, R. Tsuchikawa, J. Katoch, D. Tishler, A. M. Rao, M. Ishigami, *Acs Nano* **2011**, 5, 1594.
